# Phase-Synchronized Stimulus Presentation Augments Contingency Knowledge and Affective Evaluation in a Fear-Conditioning Task

**DOI:** 10.1523/ENEURO.0538-20.2021

**Published:** 2022-01-05

**Authors:** Elena Plog, Martin I. Antov, Philipp Bierwirth, Andreas Keil, Ursula Stockhorst

**Affiliations:** 1 Institute of Psychology, Experimental Psychology II and Biological Psychology, University of Osnabrück, D-49074 Osnabrück, Germany; 2 Department of Psychology and Center for the Study of Emotion and Attention, University of Florida, Gainesville, Florida 32611

**Keywords:** associative memory, fear conditioning, multisensory, oscillations, phase synchronization, theta band

## Abstract

Memory often combines information from different sensory modalities. Animal studies show that synchronized neuronal activity in the theta band (4–8 Hz) binds multimodal associations. Studies with human participants have likewise established that theta-phase synchronization augments the formation of declarative video–tone pair memories. Another form of associative learning, classical fear conditioning, models nondeclarative, emotional memory with distinct neuronal mechanisms. Typical fear-conditioning tasks pair a conditioned stimulus (CS) in one modality with an aversive unconditioned stimulus (US) in another. The present study examines the effects of CS–US synchronization in the theta band on fear memory formation in humans. In a fear generalization procedure, we paired one of five visual gratings of varying orientation (CS) with an aversive auditory US. We modulated the luminance of the CS and the volume of the US at a rate of 4 Hz. To manipulate the synchrony between visual and auditory input during fear acquisition, one group (*N* = 20) received synchronous CS–US pairing, whereas the control group (*N* = 20) received the CS–US pairs out of phase. Phase synchronization improved CS–US contingency knowledge and facilitated CS discrimination in terms of rated valence and arousal, resulting in narrower generalization across the CS gratings compared with the out-of-phase group. In contrast, synchronization did not amplify conditioned responding in physiological arousal (skin conductance) and visuocortical engagement (steady-state visually evoked potentials) during acquisition, although both measures demonstrated tuning toward the CS^+^. Together, these data support a causal role of theta-phase synchronization in affective evaluation and contingency report during fear acquisition.

## Significance Statement

Because of methodological limitations, examining the causal role of oscillatory synchrony in association formation has been challenging so far. Using repetitive, rhythmic sensory stimulation in a memory-related 4 Hz frequency, we examined the role of phase synchronization in fear conditioning. While synchronization improved the contingency knowledge and affective evaluation, physiological arousal and visuocortical activity were unaffected by the phase modulation. Our results represent an initial step toward establishing the causal effects of theta-phase synchronization in associative fear learning, thus improving our understanding of the neurophysiological mechanisms of fear memory encoding.

## Introduction

Phase synchronization of brain oscillations has been proposed as a mechanism supporting neuronal communication and plasticity (Fell and Axmacher, 2011). A theoretical perspective holds that the ongoing oscillatory phase reflects the excitability of a neural population and therefore determines a window for successful long-term potentiation (LTP), a cellular process underlying learning and plasticity (Lynch, 2004). Research in rodents has shown that the induction of LTP or long-term depression (LTD) critically depends on oscillatory phases and the stimulation or recording site: while LTP was induced in behaving rats when the hippocampal CA1 was stimulated at the oscillatory peak, LTD resulted from stimulation at the trough (Hyman et al., 2003). Using trace eyeblink conditioning in rabbits and recordings in the hippocampal fissure, CS presentation in the trough induced phase-locked, regular (theta) oscillations that were in turn associated with better learning, whereas CS presentation to the peak impaired regularity and learning (Nokia et al., 2015). Note that the theta phase reverses between the hippocampal fissure and the CA1 region. Since LTP requires precise timing between presynaptic and postsynaptic activation in the millisecond range (Markram et al., 1997), orchestrating activity by phase synchronization of neuronal oscillations is a potential mechanism supporting LTP. Among other oscillatory phenomena, oscillations in the theta range (4–8 Hz in primates, 4–12 Hz in rodents) and their synchronization among memory-related brain sites are linked to memory performance (Headley and Paré, 2017). Rodent research (Benchenane et al., 2010; Place et al., 2016) and human EEG studies (Weiss and Rappelsberger, 2000; Summerfield and Mangels, 2005) found increased theta synchronization among brain regions during different episodic memory tasks, suggesting that theta synchronization facilitates communication (Fell and Axmacher, 2011).

Intriguingly, studies in humans have causally linked theta-phase synchronization to episodic associative memory. Repetitive, rhythmic sensory stimulation eliciting steady-state evoked potentials (Clouter et al., 2017; Wang et al., 2018) enables experimental control over response frequency in a sensory region and corresponding phase synchrony between regions (Thut et al., 2011; Herrmann et al., 2016; Hanslmayr et al., 2019). Synchronizing the oscillatory phase evoked by periodically modulated visual and auditory stimuli facilitated the encoding of an episodic audiovisual memory (Clouter et al., 2017; Wang et al., 2018), suggesting theta-phase synchronization as a mechanism for binding multisensory episodic memories. The synchronized input is assumed to increase temporally-organized neuronal firing, which in turn may result in LTP (Buzsáki, 2002; Fries, 2015).

Although LTP is best understood in the hippocampus, its associative and synapse-specific properties make it a potential mechanism for plasticity in other regions (Maren and Fanselow, 1995; Orsini and Maren, 2012; Bliss et al., 2018). For example, fear conditioning, a paradigm of associative emotional memory, involves associative plasticity within the lateral nucleus of the amygdala (Kim and Cho, 2017), but also in other structures processing the conditioned stimulus (CS) and the unconditioned stimulus (US; Herry and Johansen, 2014). Sensory information of both stimuli (typically, different modalities) converge onto the same neuronal populations in the lateral amygdala (LA; Romanski et al., 1993). Activating weak CS synapses in temporal proximity to strong US synapses initiates a cascade of cellular reorganization, strengthening CS synapses and enabling the CS to elicit fear responses (Blair et al., 2001; Orsini and Maren, 2012). Importantly, theta synchronization among medial prefrontal cortex, amygdala, and hippocampus plays a role during fear conditioning (Seidenbecher et al., 2003; Karalis et al., 2016; Taub et al., 2018; Zheng et al., 2019; for review, see Bocchio et al., 2017; Çalişkan and Stork, 2018). However, its causal role in forming CS–US associations is unknown.

The current study asked whether synchronized sensory input helps the formation of a multisensory CS–US association in aversive learning. Using rhythmic external stimulation (Clouter et al., 2017; Wang et al., 2018), we presented the visual CS and auditory US “in-phase” or “out-of-phase” in a 2-day fear conditioning procedure with generalization (five similar CS). We hypothesized that theta-band (4 Hz) synchronization of two distinct sensory systems promotes the CS–US association. Specifically, it was expected that in-phase presentation facilitates fear acquisition, whereas out-of-phase presentation prompts poor fear conditioning. Synchronizing the multisensory input is expected to orchestrate neuronal activity in the sensory cortices (so-called entrainment). If synchronization in the theta range provides a window for successful LTP, it should optimize conditions for synchronous afferent signals reaching further structures within the fear network, especially the lateral amygdala (Romanski et al., 1993; LeDoux, 2000). To assess different response systems in human fear conditioning, we measured conditioned responses in physiological arousal, affective evaluation of arousal and valence, contingency knowledge of CS and US, and visuocortical engagement.

## Materials and Methods

### Participants

The final sample comprised 40 healthy, right handed students from the University of Osnabrück (19 - 30 years, *M* = 22.2, *SEM* = 0.35; 20 women). To control for sex hormone fluctuations, female participants were included only if they used monophasic oral contraceptives (pill) and were examined between the 6th and 20th day of pill intake (i.e., in the pill-on phase). Participants were screened via self-report questionnaire and a structured interview for inclusion and exclusion criteria in a screening session that was always conducted on a different day than the actual main experiment. Students with acute or chronic physical and/or psychiatric disorders (e.g., migraine, epilepsy, cardiovascular diseases, and phobias) were not eligible. Further exclusion criteria encompassed hearing and/or uncorrected vision impairments, tinnitus, acute medication, drug abuse, average alcohol consumption exceeding 20 or 40 g ethanol/d (for women and men, respectively), and smoking more than five cigarettes per day. Volunteers were screened for post-traumatic stress disorder (PTSD) using a translated version of the Posttraumatic Stress Diagnostic Scale (Foa, 1995; Steil and Ehlers, 2000) and excluded if they met the criteria of the DSM-IV (Diagnostic and Statistical Manual of Mental Disorders, fourth edition) for PTSD. From 64 volunteers (34 women), 46 (25 women) were eligible to participate, 3 women did not appear to the appointment, and 3 volunteers (2 women) discontinued the main experiment because of the aversive nature of the conditioning paradigm, leading to our final sample of 40 participants. Within the female and male subsamples, participants were randomly assigned to one of two groups, the in-phase or out-of-phase group, with the same number of men and women in each group (in-phase group, 10 women; out-of-phase group, 10 women).

The study was approved by the ethics committee of the University of Osnabrück and conducted in accordance with the Declaration of Helsinki guidelines. Written informed consent was obtained from all participants after adequate understanding of the explained procedures. Each participant was free to choose between participation credits (four credits) or a corresponding amount of money (32 €) for finishing the screening and day 1 and 2 of our conditioning procedure.

### Experimental design and stimuli

**Figure 1.**
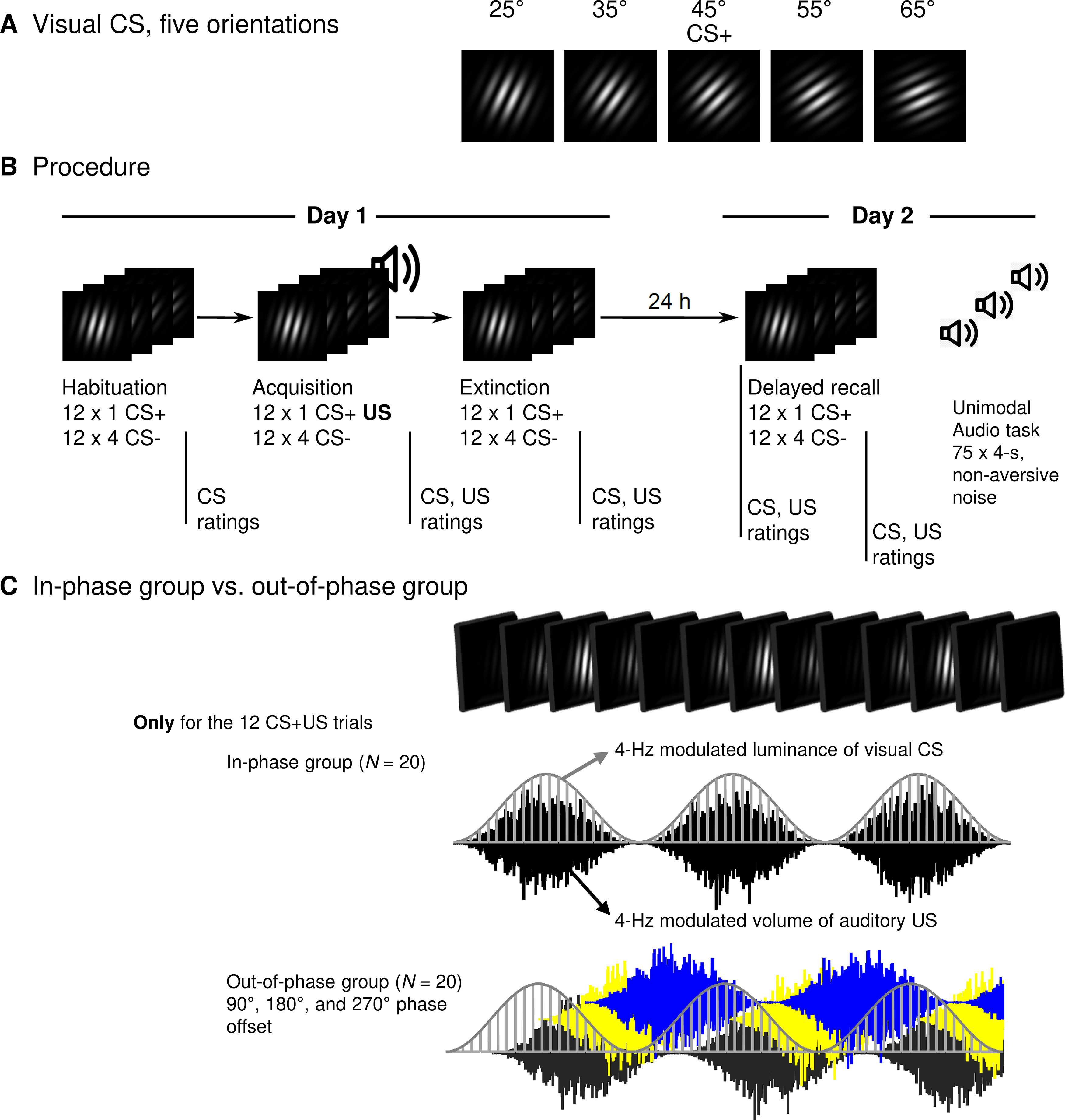
Experimental design: stimuli, procedure, and the operationalization of the in-phase group versus the out-of-phase group. ***A***, Gabor gratings used as CSs. The 45° grating served as CS^+^ (paired with the US during acquisition). The other four served as CS^–^ (never paired with the US). The luminance of each CS was sinusoidally modulated at 4 Hz. The US was a broadband white noise, amplitude modulated at 4 Hz and presented at a maximum of 96.5 dB(A). ***B***, Fear-conditioning procedure with the learning phases habituation, fear acquisition, and extinction (day 1) and delayed recall (day 2). Each CS grating was presented 12 times in each learning phase. The US was only presented during fear acquisition (12 times coterminating with the CS^+^). At the end of day 2, the unimodal audio task comprised 75 presentations of the 4 Hz modulated white noise (4 s each) at a nonaversive volume (maximum = 70.4 dB(A)]. Vertical lines below the timeline indicate the rating time points. Extended Data Figure 1-1 shows the specific trial orders 1 and 2 that were used. ***C***, Operationalization of the in-phase group versus the out-of-phase group. Fear conditioning for both groups was identical to the only exception that the in-phase group received the 12 CS^+^ US pairings during acquisition without a phase shift (0°) and the out-of-phase group received the CS^+^ US pairings with phase shifts of 90°, 180°, and 270° (four trials each). In ***C***, the top row shows a simplified depiction of a CS changing luminance at 4 Hz for 750 ms. The bottom part of ***C*** shows the first 750 ms of an overlapping CS^+^ US presentation for the two groups. The light gray curve shows the luminance of the CS^+^ (each vertical line shows one step following the 85 Hz refresh rate of the monitor). The black (0° phase shift), dark gray (90°), yellow (180°), and blue (270°) graphs show a downsampled representation of the 4 Hz modulated, white noise US.

We used a 2-day fear conditioning procedure, including habituation, acquisition, immediate extinction on day 1, and a 24 h delayed recall on day 2 (Fig. 1*B*). Our study comprised a 5 × 2 mixed factorial design within each learning phase, with five CS orientations of the below characterized Gabor gratings (25°, 35°, 45°, 55°, and 65°) as the within-subject factor and synchronization (in-phase, 0° phase shift; vs out-of-phase, 90°, 180°, and 270° phase shift); as the experimental between-subject factor.

10.1523/ENEURO.0538-20.2021.f1-1 Figure 1-1 Table of trial lists 1 and 2 for CS presentation order within each learning phase. The table shows the sequential order of CS presentation across the 60 trials of each learning phase. CS were Gabor gratings differing only in orientation (orientation degrees are shown in the second to last columns). The first column (Trial) shows the sequential number (e.g., trial 2 was the second CS seen by a participant in the specified learning phase). Each participant within the in-phase and out-of-phase groups was randomly assigned to receive stimuli according to list 1 or 2. Assignment to list 1 and 2 was balanced across groups. Download Figure 1-1, TIF file.

Five high-contrast, black-and-white Gabor gratings (i.e., a sinusoid grating filtered with a Gauss function) with a low spatial frequency served as the visual CS. The five CS differed only in orientation (25°, 35°, 45°, 55°, and 65°, relative to vertical 0°; Fig. 1*A*). The CSs were presented for 5 s centrally on a dark gray background (100% black setting on the monitor). During the presentation, the experimental chamber was lit only by the CS on the screen. Technical failure forced us to exchange the monitor from a 19 inch (model P911, Acer) to a 17 inch (model CPD-E220E, Sony) cathode ray tube (CRT) after examining the first 12 participants. We matched stimulus properties as closely as possible with the new monitor. The relevant parameters were comparable: 85 Hz refresh rate, low spatial frequency (0.96 vs 0.81 cycles/°), large central CS presentation (5.70° vs 5.73° visual angle), and high contrast (96% Michelson for both monitors).

As the US, we used a 2 s, broadband white noise (20 Hz to 22 kHz, 44,100 bits/s, 16 bits/sample), presented binaurally at a maximum of 96.5 dB(A) over two loudspeakers positioned 0.7 m left and right behind the participant. For an additional unimodal audio task (at the end of session on day 2), we presented the same white noise for 4 s at a nonaversive sound-pressure level with a maximum of 70.4 dB(A).

The intensity of the visual CS, the aversive auditory US, and the nonaversive auditory noise (unimodal task) was modulated at 4 Hz (see also Clouter et al., 2017). The luminance of the visual CS was sinusoidally modulated in 4 Hz, where luminance changed at each screen refresh, resulting in 21 steps per cycle (0–100% luminance). The amplitude of the auditory signal was sinusoidally modulated (0–100%) in 4 Hz by multiplying the signal vector with a 4 Hz sine wave at the native 44.1 kHz audio sampling rate. Presentation of each 4 Hz modulated stimulus (auditory and visual) always started at 0% intensity, increasing to 100% in the first half cycle.

### Conditioning procedure

Our procedure included habituation, acquisition, and immediate extinction on day 1, as well as a 24 h delayed recall on day 2 (Fig. 1*B*). During habituation, each 5 s, 4 Hz modulated CS was presented 12 times in pseudorandom order. Before acquisition, participants were instructed that only one of the 5 CS orientations will be followed by an aversively loud noise, without specifying which orientation. During the acquisition phase, each 4 Hz modulated CS was again presented 12 times. However, the 45° CS (CS^+^) was always paired (12 times) with the 2 s, 4 Hz modulated aversive noise US (reinforcement rate, 100%), while the other orientations were not (25°, 35°, 55°, and 65° gratings as CS^–^; Fig. 1*B*). Previous work has used this same generalization paradigm, with 45° gratings serving as CS^+^, while also establishing that there are no systematic preconditioning differences between different grating orientations on the measures used here (McTeague et al., 2015). Previous work has also demonstrated that conditioning is seen across orientations and with counterbalancing (Moratti and Keil, 2005). Together, to facilitate interpretation and comparison with prior work, this led us to adopt a fixed contingency between 45° and the US. Each CS presentation was prolonged for the duration of the US, adding 2 s (i.e., 7 s duration for the CS^+^ and the CS^–^ gratings during acquisition). For the 45° CS^+^, the last 2 s of visual CS overlapped with the auditory US presentation.

Since retinal phototransduction was shown to be slower than auditory transduction (∼50 ms for visual stimuli vs 10 ms for auditory stimuli; Lennie, 1981; King and Palmer, 1985), the onset of the auditory US had a 40 ms delay relative to the CS onset (Clouter et al., 2017). The slower transduction of visual stimuli is also in line with recordings in the amygdala after visual versus auditory stimulation. The earliest activity occurred between 40 and 80 ms (up to 316 ms, depending on the pathway to the amygdala that differs in length) after visual stimulation (Luo et al., 2010; Silverstein and Ingvar, 2015 for review, see McFadyen et al., 2017). In contrast, auditory information was recorded as early as 10–40 ms in single units of anesthetized as well as freely moving rats (Romanski et al., 1993; Quirk et al., 1995). This temporal difference in processing from receptors to the afferent neurons in the CNS has to be considered when entraining the brain with multisensory information in a synchronous way. Thus, adjusting for a temporal delay in one modality is necessary to achieve theta synchronization of visual CS and auditory US in the sensory cortices and facilitating synchrony of both inputs on the LA. CS presentation followed one of two random sequences (Extended Data Fig. 1-1), with trial order constrained to not allow more than two consecutive CSs of the same orientation. Additionally, the acquisition phase started with a booster sequence, where five of the first seven trials were CS^+^–US pairings.

The following immediate extinction, and the 24 h delayed recall phase comprised only CS presentations (12 times each, no US), as in the habituation phase. The aversive US was not presented at any point except in the 12 CS^+^–US pairings in the acquisition phase. However, neither before immediate extinction learning nor before 24 h delayed recall, participants were informed that no US will occur in the following stimulation phase.

Between the end of one CS presentation and the beginning of the next one, a black screen was shown for 3–5 s (random from a uniform distribution) during learning phases (1.5–3 s during the unimodal audio task), followed by a white fixation cross at the center of the screen for 1.5 s, resulting in an intertrial interval (ITI) between 4.5 and 6.5 s.

In accordance with the study by Clouter et al. (2017), the 12 pairings of the 4 Hz modulated CS^+^ and US were realized with either 0° (i.e., 0 ms) phase shift (in-phase group), or 90° (62.5 ms), 180° (125 ms), and 270° (187.5 ms) phase shifts for four USs each (out-of-phase group; Fig. 1*C*). Accounting for the 40 ms delay between rapid auditory and later visual processing times, input with a phase lag of 0° causes phase-synchronized cortical activity in the visual and auditory cortex (Clouter et al., 2017). This synchronized activity at the primary cortices is expected to increase the synchronized afferent signals reaching the amygdala, where CS–US convergence occurs, hence supporting associative plasticity in the lateral amygdala (Blair et al., 2001; Bocchio et al., 2017). In contrast, phase lags between 90° and 270° (i.e., timing shifts of 62.5–187.5 ms) should result in a suboptimal level of excitability and therefore decrease the likelihood of synaptic changes.

The experiment was conducted in an electromagnetically shielded and sound-attenuated experimental chamber, where participants were seated in a comfortable chair positioned centrally in front of the monitor. The experiment, including all stimuli, was created in MATLAB (version 2019b; RRID:SCR_001622) using the Psychophysics Toolbox (RRID:SCR_002881; Brainard, 1997; Kleiner et al., 2007).

### Sensory stimulation validation

**Figure 2.**
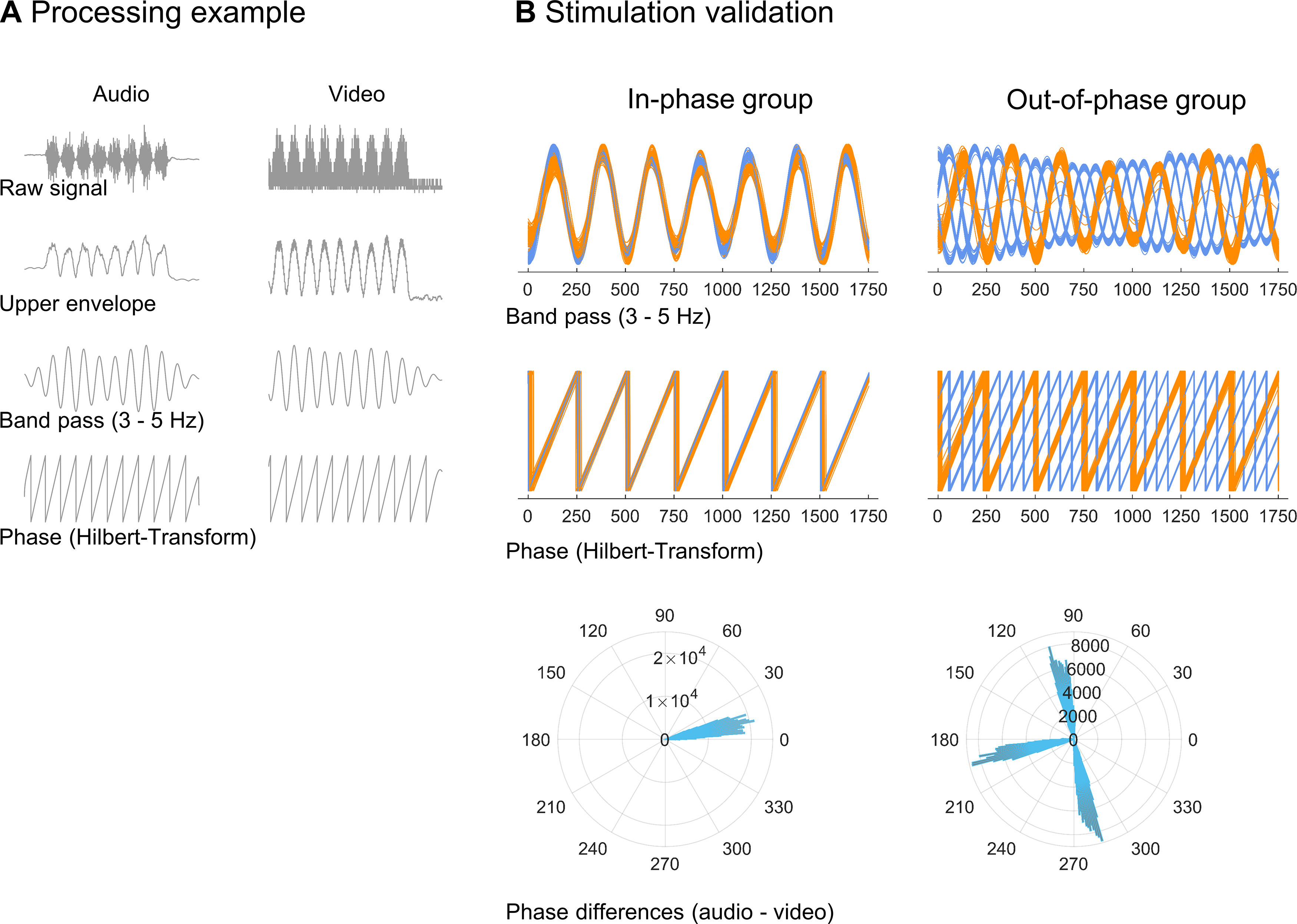
Processing steps and validation of in-phase versus out-of-phase stimulation. ***A***, Processing example (one trial of one participant) of our audio (microphone in front of the participant’s speakers) and video signal (photodiode attached to the participants’ monitor). Data were segmented relative to the onset of a US (i.e., 12 segments per subject). Before analysis, video data were shifted 40 ms forward in time to account for the 40 ms time shift programmed into the stimulus presentation. Data were rectified, bandpass filtered between 3 and 5 Hz, and subjected to a Hilbert transform. Instantaneous phase information at 4 Hz was extracted from the imaginary part of the analytic signal. ***B***, Visualization of in-phase (left column) and out-of-phase (right column) CS^+^–US stimulation for all CS^+^–US trials and all participants (12 × 20 trials per group). Each thin orange line shows the video signal of one participant and one trial. Each thin blue line shows the audio signal (one participant and trial). In ***B***, the top rows show bandpass-filtered data; the middle row shows the extracted phase information; and at the bottom, polar histograms show the clustering of all phase differences per group.

To validate the temporal fidelity of the stimulation, we analyzed data from two sources: a photograph diode (photograph sensor; Brain Products) attached to the participant’s monitor; and a microphone (built into a StimTrak, BrainVision) positioned in front of the participant’s speakers. Both signals were recorded at 1000 Hz with a recorder (BrainVision). The photograph diode was placed over the upper right corner of the CRT monitor where a smaller version of the Gabor gratings appeared during the same monitor refresh cycle (and far outside of the CS presentation area) in the same sinusoidal luminance modulation as the original CS gratings. Pilot studies using photograph sensors at both the upper corner (small-test Gabor) and the central screen (actual CS grating) showed excellent synchrony of both stimuli. The onset of the central grating was consistently 0.5 refresh cycles after the onset of the miniature grating in the top left corner (i.e., ∼5.9 ms). Using an Analyzer (BrainVision), data from the photograph sensor and the microphone were segmented from −320 to 2500 ms relative to the onset of a US (i.e., 12 segments per subject) and visually inspected for artifacts. A subset of segments was excluded, as microphone data were corrupted or missing because of the failure of the StimTrack batteries (in 5 of 40 participants). For visualization, the remaining data were exported to MATLAB and rescaled from –π to +π. Further analysis was computed over 7.02 cycles of microphone and video data (1755 ms), disregarding the last cycle of audio stimulation. Video data from −40 to +1715 ms relative to US onset were used. In contrast, microphone data from 0 to 1755 ms entered analysis. This effectively shifts video data 40 ms forward in time to account for the 40 ms time shift programmed into the stimulus presentation. Microphone data were first rectified (square root of the signal squared). Both the photograph sensor and microphone channels were bandpass filtered between 3 and 5 Hz, using the *bandpass* function of the MATLAB Signal Processing Toolbox with an IIR (infinite impulse response) filter (60 dB attenuation at the edge frequencies) and a steepness of 0.95. Instantaneous phase information at 4 Hz for the audio and video signals was extracted from the imaginary part of the analytic signal after a Hilbert transform (Fig. 2*A*).

This analysis Fig. 2*B* also demonstrates that there was very little variability in the timing of sensory stimulation within a trial, as well as between trials and between participants of one group.

### Dependent variables

Steady-state visually evoked potentials (ssVEPs; via EEG), skin conductance responses (SCRs), and subjective ratings served as dependent outcomes. Further, horizontal and vertical eye movements were recorded by electrooculography (EOG) with a bipolar BrainAmpExG Amplifier (BrainProducts) to detect and eliminate artifacts in the EEG recordings.

### EEG parameters

#### EEG recording and preprocessing

A 64-channel EEG was recorded on both days with two 32-channel BrainAmp DC amplifiers with a resolution of 0.1 μV (Brain Products). The 64 active electrodes (Ag/AgCl, actiCAP, Brain Products) were filled with electrolyte gel (Super-Visc 10% NaCl, EasyCap) and positioned according to the extended international 10–20 system. Efforts were made to keep impedances <5 kΩ (manufacturer recommendation, <25 kΩ). FCz served as the recording reference, and AFz served as the ground. A sampling rate of 1000 Hz and a high-pass filter at 0.016 Hz were used. In addition to the EEG, EOG was recorded with four Ag/AgCl electrodes (Ø, 4 mm) to control for eye movements. Two electrodes were placed on the lateral canthus of each eye for horizontal movements, and two electrodes were placed infraorbital and supraorbital, in line with pupil of the right eye, for vertical movements. An electrode on the forehead was attached as the ground.

Offline preprocessing was done with Analyzer 2 Software (version 2.1.2.327; BrainVision). Raw data were bandpass filtered between 1 and 100 Hz using Butterworth (zero phase shift) filters with a 3 dB low cutoff at 1 Hz (time constant, 0.1592; order 8) and a 3 dB high cutoff at 100 Hz (order 4). Additional 50 and 100 Hz (bandwidth, 1 Hz; order 4) notch filters were applied to eliminate line noise. Data were segmented from −1250 to 7500 ms relative to a CS onset, and an ocular correction independent component analysis (ICA), as implemented in BrainVision Analyzer, was applied. After visual inspection of the resulting factors and factor topographies, factors related to horizontal and vertical eye movements, blinks, as well as strong cardiac or muscular artifacts were removed from the reconstructed data. ICA-corrected data were rereferenced to an average reference, and the recording reference was reincluded in the data as a 65th channel at position FCz. The segments were cut to an interval between −1000 and 5000 ms relative to CS onset. With this segmentation, the US intervals were excluded from further analyses to avoid contamination of our EEG data. After another visual inspection, we rejected segments with remaining artifacts. On average, 3.93 segments were rejected per participant (0–15 rejected of 240 segments for each participant). Data were downsampled to 512 Hz, in accordance with the findings of Clouter et al. (2017), and were exported to MATLAB (MathWorks; RRID:SCR_00162). To increase spatial specificity, reduce volume conduction effects, and obtain reference-free data, we conducted a scalp current source density (CSD) transform (Junghöfer et al., 1997). The CSD values (as estimates of cortical surface potentials) are represented on a sphere, approximating a cortical surface. For scalp-level analyses and topographical mapping, the CSD was projected back onto the original electrode space. Analyses were performed on CSD-transformed data, and CSD data are shown throughout the figures.

#### Validation of visual and auditory entrainment (unimodal)

To validate the visual cortical entrainment at 4 Hz, we first averaged CSD-transformed data over all habituation trials at each sensor of a participant in the time domain [disregarding CS orientations (i.e., averaging 5 × 12 trials per subject)]. Habituation trials were not only strictly unimodal visual stimulation but preceded any pairing of the CS with the aversive US. To avoid early event related potentials entering the frequency domain analysis, Fourier transform was applied on data from 750 to 5000 ms (i.e., containing 17 cycles of 4 Hz) post-CS onset. These data were windowed with a cosine-square window (20 point rise/fall) and subjected to a discrete Fourier transform (MATLAB) with a frequency resolution of 0.24 Hz. We extracted the absolute values of the Fourier coefficients at 4 Hz and transformed the resulting power values to signal-to-noise ratios (SNRs), using the average of five frequency bins <4 Hz and four frequency bins >4 Hz.

To validate auditory entrainment, we used the unimodal audio task at the end of day 2, as it reflected 4 Hz unimodal auditory stimulation, without concurrent visual stimulation. EEG data from the audio-only task were subjected to the same preprocessing pipeline as CS-related data. As for the visual unimodal data described above, CSD data were segmented (here from −1000 to 4100 ms, relative to audio stimulus onset) and averaged per participant and sensor over the 75 audio-only trials. Fourier transform was applied on windowed data (cosine-square, 20 point rise/fall) starting from 500 to 4000 ms after audio onset (i.e., containing 14 cycles of 4 Hz), resulting in a frequency resolution of 0.29 Hz. Like for the visual stimulation, we converted the power at 4 Hz to SNRs, using the average of the five frequency bins below and four above the frequency of interest as noise estimates.

**Figure 3.**
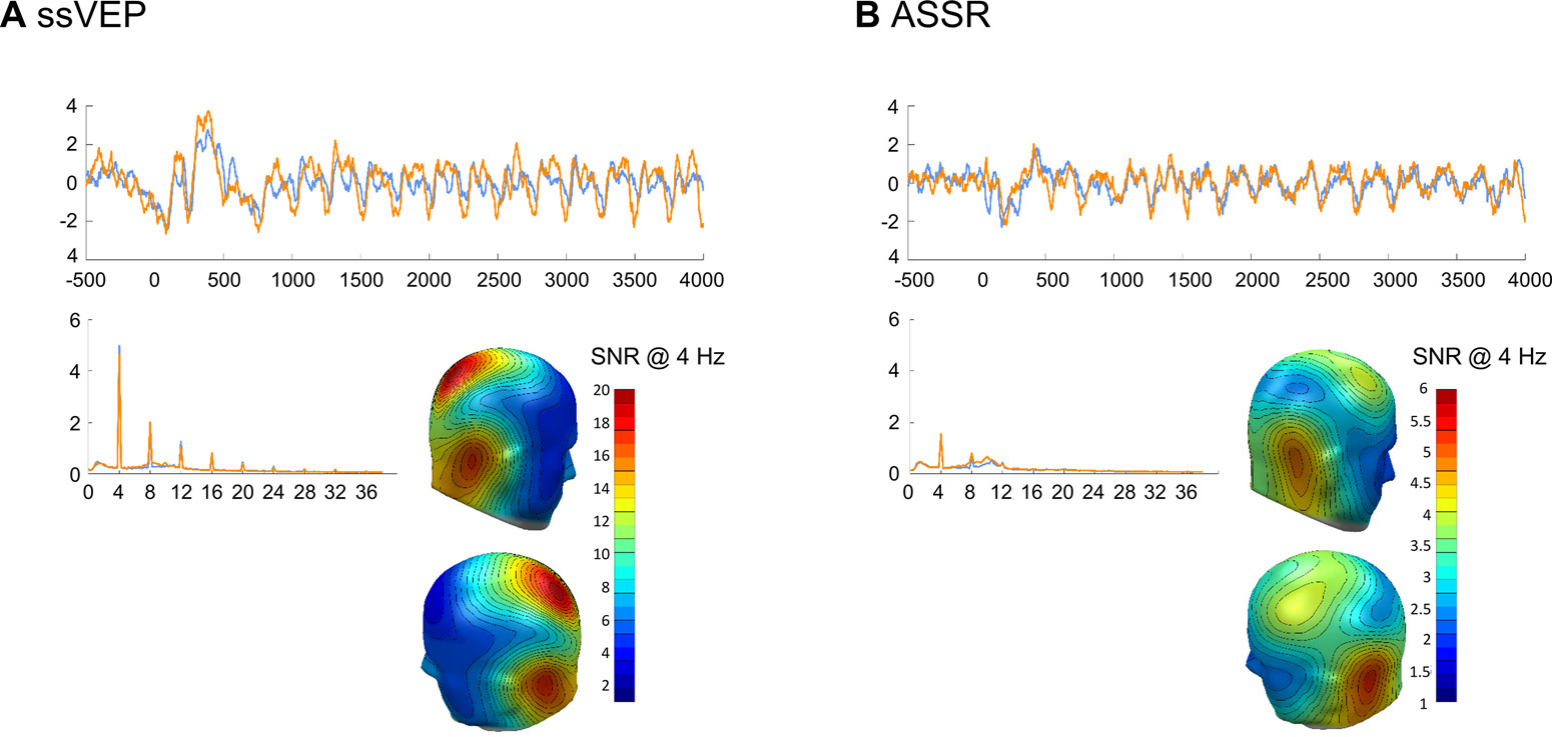
ssVEP and the auditory steady-state response 4 Hz signal in the time domain and frequency domain, as well as the scalp distribution of the 4 Hz signal. ***A***, ***B***, The signal-to-noise ratio, averaged over all 40 participants (i.e., regardless of factor group) is presented for the visual (***A***) and auditory (***B***) 4 Hz stimulation. Orange lines show averaged data from participants of the in-phase group, blue lines show data from the out-of-phase group.

Figure 3 shows the scalp distribution of the 4 Hz SNR averaged over participants (*N* =* *40), for the visual (Fig. 3*A*) 4 Hz stimulation and the auditory 4 Hz stimulation (Fig. 3*B*). The average topographies are consistent with typical visual and auditory steady-state evoked potential at 4 Hz, respectively. Specifically, the relatively low driving frequency of 4 Hz has traditionally been shown to prompt larger spread of the ssVEP topography, reflective of longer individual stimulation cycles, which allow spreading across the visual hierarchy (Skrandies, 2007). However, the topographies of the 4 Hz EEG signal showed some variation between subjects. Therefore, for subsequent analyses in the frequency domain, including single-trial analyses of CS-related activity, we selected the six individual sensors for each participant showing the highest SNR at 4 Hz.

#### ssVEP single-trial analysis

We conducted a single-trial analysis to be able to show the temporal evolution of visual cortical engagement over the course of learning trials. For single-trial analysis, we used data segments between −1000 and 5000 ms, relative to CS onset as 0 (in sample points at 512 Hz sample rate, this is 1–3072 sample points with zero being sample point 512). First, we sampled it up from 512 to 1536 Hz. Upsampling the data ensured an integer number of sampling points per one cycle for the 4 Hz as well as its harmonics (up to 16 Hz). At 1536 Hz, one cycle of the driving frequency (4 Hz) is 384 samples (instead of 128 at 512 Hz). By subtracting the mean of the 1000 ms prestimulus interval, the data were baseline corrected. The power extraction of single trials was based on the analysis window between 750 and 5000 ms (relative to 0 ms = CS onset). Over this analysis window, a moving average procedure was conducted. We obtained averages by shifting a window with a length of four cycles of the frequency of interest (i.e., 4 Hz) across the detrended data segments in steps of one cycle and averaging the contents of the window with each step (12 steps, last four-cycle step starting at 3000 ms after CS onset). We then transformed the single-trial estimates from the time into the frequency domain using discrete Fourier transform (DFT) and extracted the power at the driving frequency as the absolute of the Fourier coefficients, normalized by the length of FFT (here, 1536 sample points).

Interindividual variance in response strength and pre-experimental bias was corrected by calculating a habituation ratio for each CS (via division by habituation mean over all 60 trials of each participant, disregarding the different CS orientations), with values >1 describing an enhancement and <1 describing a decrease of ssVEP power compared with habituation. In addition, single-trial data were smoothed with a moving average along the 12 trials (5 point symmetrical, shrinking at the end points) within each learning phase and CS orientation (5 orientations × four learning phases with 12 trials each). For plots showing the temporal evolution of ssVEP over trials, we pooled data over sensors as the average of the individually defined six maximal SNR sensors for each participant. The individual sensors entering this six-sensor cluster were defined as the six sensors showing the highest SNR at 4 Hz during habituation trials for a participant [see above, Validation of visual and auditory entrainment (unimodal)]. Of note: while single-trial data are interesting and informative, we have no prior evidence allowing us to formulate specific hypotheses about group differences (in phase vs out of phase) in the temporal dynamics of ssVEP. Therefore, these data were averaged over all trials of a learning phase before statistical testing for group effects.

#### Skin conductance responses and electrocardiography, and blood pressure

In addition to ssVEP power tunings toward specific CS gratings, we used SCRs as a common measure of learning-induced changes in physiological arousal to the CS. As our laboratory is configured for stress-associated questions by default, we also recorded electrocardiography (EKG) and blood pressure (BP) as control parameters only. EKG and BP will not be reported in the Results section. We used a Brainamp ExG amplifier (Brain Products) and a 0.5 V constant voltage coupler to record SCRs with a sampling rate of 1000 Hz and a resolution of 0.0061 μS. We attached two Ø 10 mm (inner diameter) electrodes, filled with 0.05% NaCl paste (TD-246) on the thenar and hypothenar of the left hand (nondominant) of each participant (Boucsein et al., 2012). No additional filters were applied. Data were downsampled to 200 Hz in BrainVision Analyzer 2.1 and exported to MATLAB. Responses with an onset latency between 1 and 4 s and a minimum amplitude of 0.02 μS were automatically scored using Ledalab (Benedek and Kaernbach, 2010). If more than one response met the criteria, single responses were summed up. Responses that did not meet the criteria were scored as zero. After Ledalab scoring, we used an additional visual inspection of heat maps of single trials and corrected 42 values (of 240 trials × 40 participants = 9600 total values) that were overscored or underscored by Ledalab. To further correct for interindividual differences and push distribution toward normal, we calculated *z*-values using the means and SDs of CS and US responses of all learning phases (habituation, acquisition, immediate extinction, and delayed recall) per participant. In accordance with ssVEPs, single-trial data were smoothed with a moving average along the 12 trials (5 point symmetrical, shrinking at the end points) within each learning phase and CS orientation (5 orientations × 4 learning phases with 12 trials each). The *z*-standardized SCRs were then averaged across the 12 trials of each learning phase, and the averages were used in all statistical analyses.

For the recording of EKG, we positioned three Ø 8 mm (inner diameter) electrodes (filled with 5% NaCl EKG paste, GE Medical Systems Information Technologies) under the right collarbone, the left shinbone, and (as ground electrode) on the right shinbone.

Systolic and diastolic BP were measured at discrete measurement points using a semiautomatic electronic sphygmomanometer (bosotron 2, Bosch + Sohn). Therefore, an inflatable cuff was placed around the left upper arm, with the sensor plate positioned over the brachial artery at heart level.

### Subjective ratings: valence, arousal, and US expectancy

A paper–pencil version of the 9 point pictorial Self-Assessment Manikin (SAM; Bradley and Lang, 1994) scale was used to evaluate each CS orientation for its valence (from negative to positive) and arousal (from excited to calm). Ratings were conducted after habituation, acquisition, and immediate extinction, as well as before and after 24 h delayed recall. In addition, we asked the participants to rate their expectancy that a US occurs with the depicted grating with answers ranging from −5 (very certain, no), to 0 (uncertain), to 5 (very certain, yes). Except for after habituation, paper−pencil US expectancy ratings were conducted together with our SAM ratings.

### Overall procedure

The study covered the following two parts: the screening session, lasting ∼1 h, explaining the general procedure of the main session, testing for inclusion and exclusion criteria and obtaining informed consent (for description, see the Participants section); and the main conditioning study. Screening and the main study were scheduled on different days.

### Main conditioning study

The main session was conducted on two consecutive days, starting at 10:00 A.M., 2:00 P.M., or 5:30 P.M. The duration of day 1 and day 2 of the main session were 2 and 1 h, respectively. At the beginning of day 1 and day 2, we attached EEG, EKG, EOG, and SCR electrodes, and positioned the inflatable cuff for BP measures. Habituation, acquisition, and immediate extinction took place on day 1, while a 24 h delayed recall took place on day 2. After each learning phase on day 1 (i.e., after habituation, acquisition, and immediate extinction) as well as before and after delayed recall on day 2, resting periods, SAM and US expectancy ratings (except after habituation, where SAMs were conducted without US expectancy ratings, since no US has occurred), and EKG, SCR, as well as BP measures were performed (Fig. 1*B*).

Before starting the computer task, we read the standardized “general information about the experiment,” including a description of the procedure and the stimuli we were about to present. Subjects were instructed to sit comfortably and avoid any movements (except eye blinking) for the entire duration of the computer tasks and the explicitly announced measurement periods. During the resting phases, subjects were encouraged to move carefully to avoid the detachment of electrodes. At the beginning of habituation, subjects were asked to fixate on an upcoming white cross in the center of the screen followed by some black and white “flickering” gratings. Before acquisition, we informed the participants that a loud flickering noise would be presented with only one of the gratings. However, we did not specify which of the five orientations would predict the aversive noise. Before immediate extinction (day 1) and delayed recall (day 2), participants were asked to remember the instructions, without informing them that no aversive noise would be presented.

### Statistical analysis

**Figure 4.**
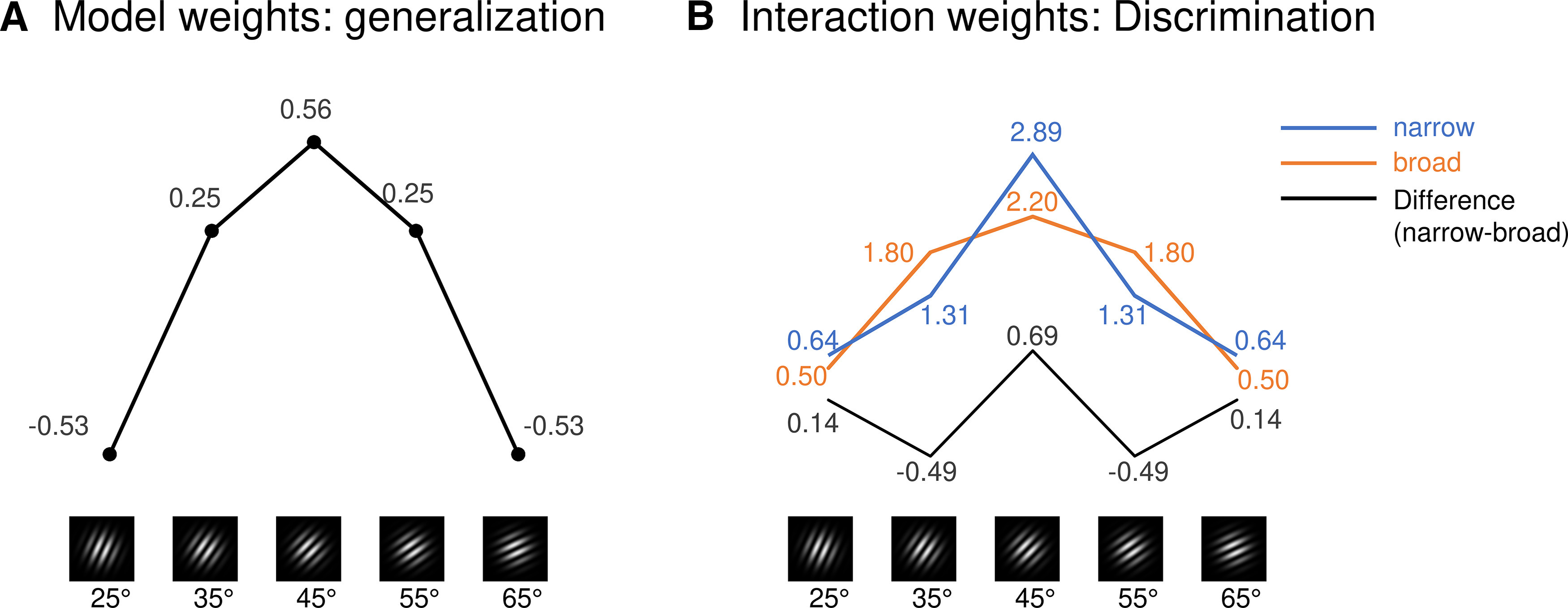
Contrast weights. ***A***, Generalization weights to test the fit for a generalized fear response toward the CS^+^ and neighboring CS^–^ orientations, independent of the factor group. ***B***, Contrast weights (discrimination) to test the group × orientation interaction. The weights shown for a narrow (blue) and broad (orange) generalization pattern are just examples that if subtracted (narrow – broad) produce the exact discrimination weights we used for the group × orientation interaction contrast (numbers in black font, 0.142, −0.498, 0.694, −0.498, 0.142), resembling a Mexican Hat (black line). For better readability, contrast weights in the graphs A and B are inserted with 2 decimals.

We submitted each of the memory outcome measures (i.e., US expectancy ratings, affective valence and arousal ratings, SCRs, and ssVEPs) to a 5 × 2 repeated-measures ANOVA, conducted using SPSS software (version 26.0; SPSS). The mixed ANOVA included the within-subject factor orientation (i.e., the five CSs: 25°, 35°, 45°, 55°, and 65°) and the between-subject factor group (i.e., in-phase group, 0° phase offset; vs out-of-phase group, 90°, 180°, and 270° phase offset). To test for the expected form of the orientation effect independent of group, we conducted a custom contrast for generalization, using contrast weights adapted from prior studies (Fig. 4*A*; generalization weights: −0.529, 0.247, 0.564, 0.247, and −0.529).

As this is the first attempt to compare a synchronized versus nonsynchronized condition with a fear generalization design, we hypothesized that group differences may manifest in one of the following three possible ways: (1) synchronized CS–US presentation may lead to major increases in CS responding not limited to the CS^+^ (this would be evident in a main effect group in the ANOVA); (2) synchronized CS–US presentation may dramatically change the pattern of responding over the five different CSs (this could be evident in an orientation × group interaction in the ANOVA); and (3) finally, synchronized CS–US presentation may alter the width of the generalization curve. This could optimize learning, resulting in a narrower generalization and thus better discrimination among the five CSs, without changing overall response levels or dramatically changing the response pattern. ANOVA interactions would not be able to detect this. Therefore, we designed a custom contrast for the group × orientation interaction, using the LMATRIX command for contrast coefficient matrices in SPSS. We obtained the contrast weights by subtracting a broader generalization profile (Fig. 4*B*, orange line and font) from a narrower generalization profile (Fig. 4*B*, blue line and font), resulting in a form resembling a “Mexican Hat” (weights: 0.142, −0.489, 0.694, −0.489, and 0.142). We expected group differences to manifest during (or directly after) acquisition. However, to explore the longevity of potential group effects, we repeated our 5 × 2 ANOVA and the custom Mexican Hat group × orientation contrast for immediate extinction and delayed recall on day 2.

## Results

### Phase synchronization causes a better discrimination between CS^+^ and neighboring CS^–^ gratings in the US expectancy ratings

**Figure 5.**
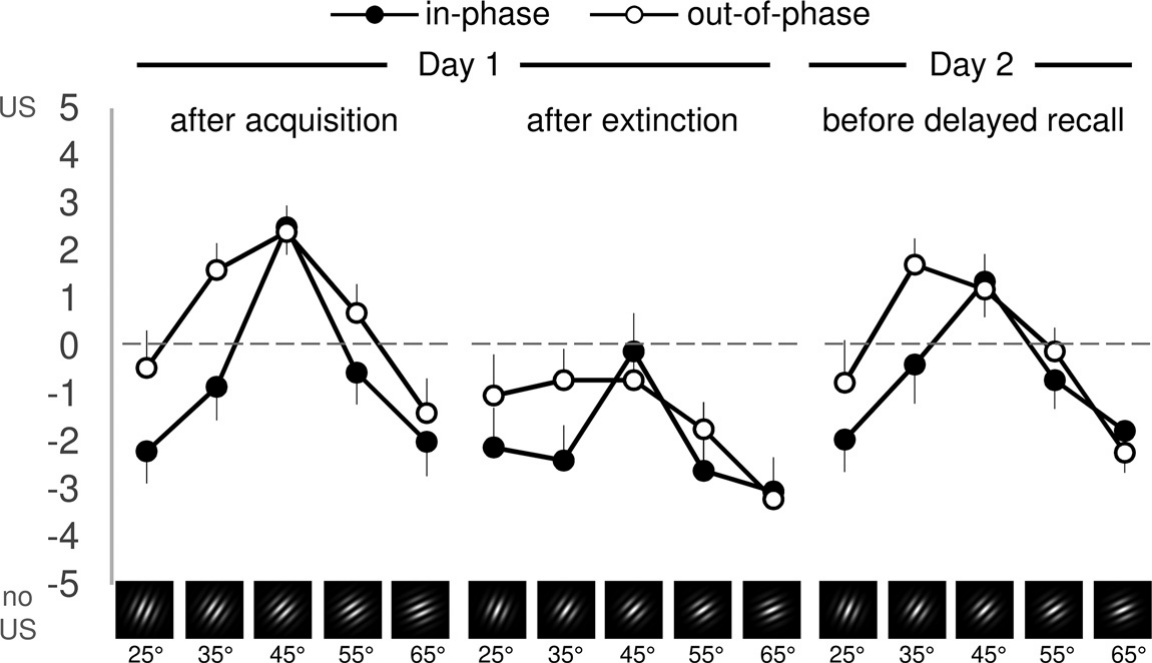
US expectancy ratings separated for each measurement point: after acquisition, after extinction on day 1, and before delayed recall on day 2 in the in-phase and the out-of-phase groups. US expectancy was rated per CS on scale ranging from −5 (very certain, no US after this CS) over 0 (uncertain) to 5 (very certain, a US will follow this CS). Each data point presents the mean US expectancy rating for each CS orientation (averaged over participants per group and measurement point), error bars show 1 SEM. Extended Data Figure 5-1 shows discrimination indices (CS^+^ minus the weighted average of all CS^–^) and estimation statistics for US expectancy ratings. For transparency, Extended Data Figure 5-2 shows discrimination indices that result when subtracting the unweighted average of the CS^–^ from the CS^+^.

We found an effect of orientation on US expectancies collected immediately after the CS^+^ was repeatedly aversively reinforced during acquisition (*F*_(3,109)_ = 12.491, *p *=* *6.764E-7, partial η^2^ = 0.247; Table 1, a). The resulting pattern reflected generalization around the CS^+^ (Fig. 5), with the CS^+^ and the most similar gratings receiving the highest US expectancy scores (generalization contrast fit: *F*_(1,38)_ = 28.360, *p *=* *0.000005, partial η^2^ = 0.427, Table 1, b). In addition, data revealed a main effect of group (*F*_(1,38)_ = 7.310, *p *=* *0.010, partial η^2^ = 0.161; Table 1, c), but no group × orientation interaction (Table 1, d). Here, the out-of-phase group showed broader generalization of the US expectancy ratings, while the in-phase group had a narrower generalization pattern with more discrimination between the CS^+^ and the four CS^–^ (Fig. 5). This was supported by a significant orientation × group interaction in the form of a Mexican Hat (*F*_(1,38)_ = 4.796, *p *=* *0.035, partial η^2^ = 0.112; Table 1, e). As a comprehensive index of CS discrimination (i.e., CS^+^ vs average of all CS^–^), we calculated discrimination indices by subtracting the weighted average of CS^–^ responses from the CS^+^ responses (Extended Data Fig. 5-1). To account for the fact that the 35° and 55° CS^–^ orientations only differ from the CS^+^ by 10° and are thus harder to discriminate, these orientations were multiplied with a weight of 0.33[…] before averaging. The more dissimilar orientations (25°, 65°) differ by 20° from the CS^+^ and are easier to discriminate. Therefore, these two were weighted with 0.166[…] (i.e., half of the weight of the more similar orientations). Although the CS^–^ weights account for the perceptual difference, they are not directly derived from a psychophysics curve. Extended Data Figure 5-1 depicts estimation statistics for the discrimination indices within each learning phase by presenting individual values as well as the effect sizes (Hedge’s *g*) as a bootstrap 95% confidence interval (5000 samples; Ho et al., 2019). To increase transparency, Extended Data Figure 5-2 shows the same for a discrimination index computed with the unweighted average of the four CS^–^ values.

**Table 1:** Summary of statistical analyses

	Data structure	Type of test	Effects	Statistic	*p* Value	Effect size
US expectancy						
Acquisition						
a	Normal	ANOVA	ME: o	*F*_(3,109)_ = 12.491	6.764E-7	*η^2^*_p_ = 0.247
b	Normal	ANOVA	GEN	*F*_(1,38)_ = 28.360	0.000005	*η^2^*_p_ = 0.427
c	Normal	ANOVA	ME: g	*F*_(1,38)_ = 7.310	0.010	*η^2^*_p_ = 0.161
d	Normal	ANOVA	o × g INT	*F*_(3,109)_ = 1.133	0.338	*η^2^*_p_ = 0.029
e	Normal	ANOVA	MEX	*F*_(1,38)_ = 4.796	0.035	*η^2^*_p_ = 0.112
Extinction						
f	Normal	ANOVA	ME: g	*F*_(1,38)_ = 0.621	0.436	*η^2^*_p_ = 0.016
g	Normal	ANOVA	o × g INT	*F*_(3,113)_ = 1.363	0.258	*η^2^*_p_ = 0.035
h	Normal	ANOVA	MEX	*F*_(1,38)_ = 6.660	0.014	*η^2^*_p_ = 0.149
Delayed recall (day 2)						
i	Normal	ANOVA	ME: g	*F*_(1,36)_ = 0.688	0.412	*η^2^*_p_ = 0.019
j	Normal	ANOVA	o × g INT	*F*_(3,100)_ = 1.172	0.323	*η^2^*_p_ = 0.032
k	Normal	ANOVA	MEX	F_(1.36)_ = 3.090	0.087	*η^2^*_p_ = 0.079
Valence and arousal						
Acquisition						
i	Normal	ANOVA_Val_	ME: o	*F*_(3,96)_ = 7.756	0.000272	*η^2^*_p_ = 0.170
m	Normal	ANOVA_Aro_	ME: o	*F*_(3,100)_ = 10.928	0.000008	*η^2^*_p_ = 0.223
n	Normal	ANOVA_Val_	GEN	*F*_(1,38)_ = 12.352	0.001	*η^2^*_p_ = 0.245
o	Normal	ANOVA_Aro_	GEN	*F*_(1,38)_ = 19.587	0.000078	*η^2^*_p_ = 0.340
p	Normal	ANOVA_Val_	ME: g	*F*_(1,38)_ = 1.221	0.276	*η^2^*_p_ = 0.031
q	Normal	ANOVA_Val_	o × g INT	*F*_(3,96)_ = 1.502	0.224	*η^2^*_p_ = 0.038
r	Normal	ANOVA_Aro_	ME: g	*F*_(1,38)_ = 1.248	0.271	*η^2^*_p_ = 0.032
s	Normal	ANOVA_Aro_	o × g INT	*F*_(3,100)_ = 1.658	0.187	*η^2^*_p_ = 0.042
t	Normal	ANOVA_Val_	MEX	*F*_(1,38)_ = 9.228	0.004	*η^2^*_p_ = 0.195
u	Normal	ANOVA_Aro_	MEX	*F*_(1,38)_ = 7.325	0.010	*η^2^*_p_ = 0.162
Extinction						
v	Normal	ANOVA_Val_	ME: g	*F*_(1,38)_ = 1.810	0.186	*η^2^*_p_ = 0.045
w	Normal	ANOVA_Val_	o × g INT	*F*_(3,117)_ = 0.647	0.590	*η^2^*_p_ = 0.017
x	Normal	ANOVA_Aro_	ME: g	*F*_(1,38)_ = 0.355	0.555	*η^2^*_p_ = 0.009
y	Normal	ANOVA_Aro_	o × g INT	*F*_(3,112)_ = 0.437	0.724	*η^2^*_p_ = 0.011
Delayed recall (day 2)						
z	Normal	ANOVA_Val_	ME: g	*F*_(1,36)_ = 0.074	0.788	*η^2^*_p_ = 0.002
aa	Normal	ANOVA_Val_	o × g INT	*F*_(3,96)_ = 0.216	0.864	*η^2^*_p_ = 0.006
bb	Normal	ANOVA_Aro_	ME: g	*F*_(1,36)_ = 0.239	0.628	*η^2^*_p_ = 0.007
cc	Normal	ANOVA_Aro_	o × g INT	*F*_(3,100)_ = 0.121	0.938	*η^2^*_p_ = 0.003
SCRs						
Acquisition						
dd	Normal	ANOVA	ME: o	*F*_(3,96)_ = 14.856	3.1057E-7	*η^2^_p_* = 0.281
ee	Normal	ANOVA	GEN	*F*_(1,38)_ = 31.987	0.000002	*η^2^*_p_ = 0.457
ff	Normal	ANOVA	ME: g	*F*_(1,38)_ = 0.931	0.341	*η^2^*_p_ = 0.024
gg	Normal	ANOVA	o × g INT	*F*_(3,96)_ = 0.833	0.461	*η^2^*_p_ = 0.021
Extinction						
hh	Normal	ANOVA	ME: g	*F*_(1,38)_ = 1.170	0.286	*η^2^*_p_ = 0.030
ii	Normal	ANOVA	o × g INT	*F*_(3,117)_= 0.921	0.435	*η^2^*_p_ = 0.024
Delayed recall (day 2)						
jj	Normal	ANOVA	ME: g	*F*_(1,38)_ = 0.002	0.965	*η^2^*_p_ = 0.00005
kk	Normal	ANOVA	o × g INT	*F*_(3,116)_= 1.483	0.222	*η^2^*_p_ = 0.038
ssVEPs						
Acquisition						
ll	Normal	ANOVA	ME: o	*F*_(4,137)_ = 5.696	0.000479	*η^2^*_p_ = 0.130
mm	Normal	ANOVA	GEN	*F*_(1,38)_ = 8.447	0.006	*η^2^*_p_ = 0.182
nn	Normal	ANOVA	o × g INT	*F*_(4,137)_ = 1.042	0.384	*η^2^*_p_ = 0.027
Extinction						
oo	Normal	ANOVA	ME: g	*F*_(1,38)_ = 2.957	0.094	*η^2^*_p_ = 0.072
pp	Normal	ANOVA	o × g INT	*F*_(4,147)_ = 0.418	0.790	*η^2^*_p_ = 0.011
Delayed recall (day 2)						
qq	Normal	ANOVA	ME: g	*F*_(1,38)_ = 5.354	0.026	*η^2^*_p_ = 0.123
rr	Normal	ANOVA	o × g INT	*F*_(3,122)_ = 0.556	0.657	*η^2^*_p_ = 0.014

Table shows statistical analyses including *p* value and effect size for each memory outcome measure, separated by learning phase. For each outcome measure, we calculated repeated-measures ANOVAs with the CS orientation as the within-subject factor and the group (in-phase group vs out-of-phase group) as the between-subject factor. Successful conditioning (i.e., increased response toward the CS^+^ respective of group) was validated by the main effects of orientations (noted in the column effects as ME: o). To account for the specific symmetric generalization pattern (CS^+^ in the middle), additional generalization contrast fits were used (noted as GEN). The main effects of group (ME: g) and group × orientation interactions (o × g INT) addressed differences between in-phase and out-of-phase conditioning. Better grating discrimination versus stronger generalization across orientations are described by a Mexican Hat contrast fit for the group × orientation interactions (MEX). ANOVA, Mixed repeated-measures ANOVA; ME, main effect; o, orientation; η^2^_p_, partial η^2^; g, group; MEX, Mexican Hat contrast fit of orientation × group interaction; INT, interaction; GEN, generalization fit; Val, valence; Aro, arousal.

10.1523/ENEURO.0538-20.2021.f5-1 Figure 5-1 Weighted discrimination indices for US expectancy ratings. US expectancy ratings were first *z*-transformed within each participant using the mean and SD of all US expectancy ratings of a participant. With the *z*-transformed data we computed a weighted discrimination index per learning phase as the difference between the rating of the reinforced 45° (CS^+^) grating and the weighted average of the four CS^–^ gratings. Weights for the CS^–^ correspond to the angular difference in orientation between the four CS^–^ orientations (25°, 35°, 55°, 65°) and the CS^+^ orientation (45°). The two more similar CS^–^ orientations (±10° to the CS^+^) were weighted with 0.33[…], while the more dissimilar orientations (±20° to the CS^+^) were weighted with 0.166[…]. Data and effect sizes are shown as a Cumming estimation plot (http://www.estimationstats.com). Top row, Swarm plots show the raw discrimination indices per learning phase (each dot is the discrimination index of one participant). Group statistics are indicated to the right of each swarm as gapped lines (gap = mean, line length = 1 SD). Bottom row, Effect size estimates (Hedges’ *g*, black dots) for the three relevant comparisons (in-phase vs out-of-phase for each learning phase) and their 95% confidence interval (CI; vertical error bars). The unpaired Hedge’s *g*: for acquisition: –0.364 [95% CI, –0.981, 0.315], *p *= 0.2578; for extinction: –0.463 [95% CI, –1.089, 0.205], *p *= 0.1532; for delayed recall: –0.249 [95% CI, –0.907, 0.370], *p =* 0.4206. The 5000 bootstrap samples were taken for CI estimation; the CI is bias corrected and accelerated. The two-sided *p* values are the likelihoods of observing the effect sizes, if the null hypothesis of zero difference is true. For each permutation *p* value, 5000 reshuffles of the group labels were performed. Download Figure 5-1, TIF file.

10.1523/ENEURO.0538-20.2021.f5-2 Figure 5-2 Unweighted discrimination indices for US expectancy ratings. US expectancy ratings were *z*-transformed within each participant using the mean and SD of all US expectancy ratings of a participant. The unweighted discrimination index shown is the difference between ratings of the CS^+^ and the unweighted average of the four CS^–^ orientations. Data and effect sizes are shown as a Cumming estimation plot (http://www.estimationstats.com). See the legend of Extended Data Figure 5-1 for a detailed description of a Cumming estimation plot. The unpaired Hedge’s *g*: for acquisition:–0.306 [95% CI, –0.928, 0.375], *p *=* *0.3356; for extinction: –0.372 [95% CI, –1.021, 0.289], *p* = 0.2346; for delayed recall: –0.198 [95% CI, –0.842, 0.433], *p *= 0.5166. The 5000 bootstrap samples were taken for CI estimation; the CI is bias corrected and accelerated. The two-sided *p* values are the likelihoods of observing the effect sizes, if the null hypothesis of zero difference is true. For each permutation *p* value, 5000 reshuffles of the group labels were performed. Download Figure 5-2, TIF file.

For US expectancy ratings collected after extinction, we found no main effect of group or a group × orientation interaction (Table 1, f, g). However, even after extinction trials, the in-phase group showed a narrower generalization pattern than the out-of-phase group (Fig. 5). Mexican Hat contrast fit for the orientation × group interaction (*F*_(1,38)_ = 6.660, *p *=* *0.014, partial η^2^ = 0.149, Table 1, h). On day 2, 24 h later (Fig. 5, Extended Data Fig. 5-1, day 2 before delayed recall) we found no group differences in US expectancy ratings (no main effect group, no orientation × group interaction; Table 1, i, j), and the generalization was no longer significantly narrower in the in-phase group (Mexican Hat orientation × group interaction; Table 1, k).

### Synchronization leads to a narrower rating pattern toward the CS^+^ in valence and arousal ratings after fear acquisition

**Figure 6.**
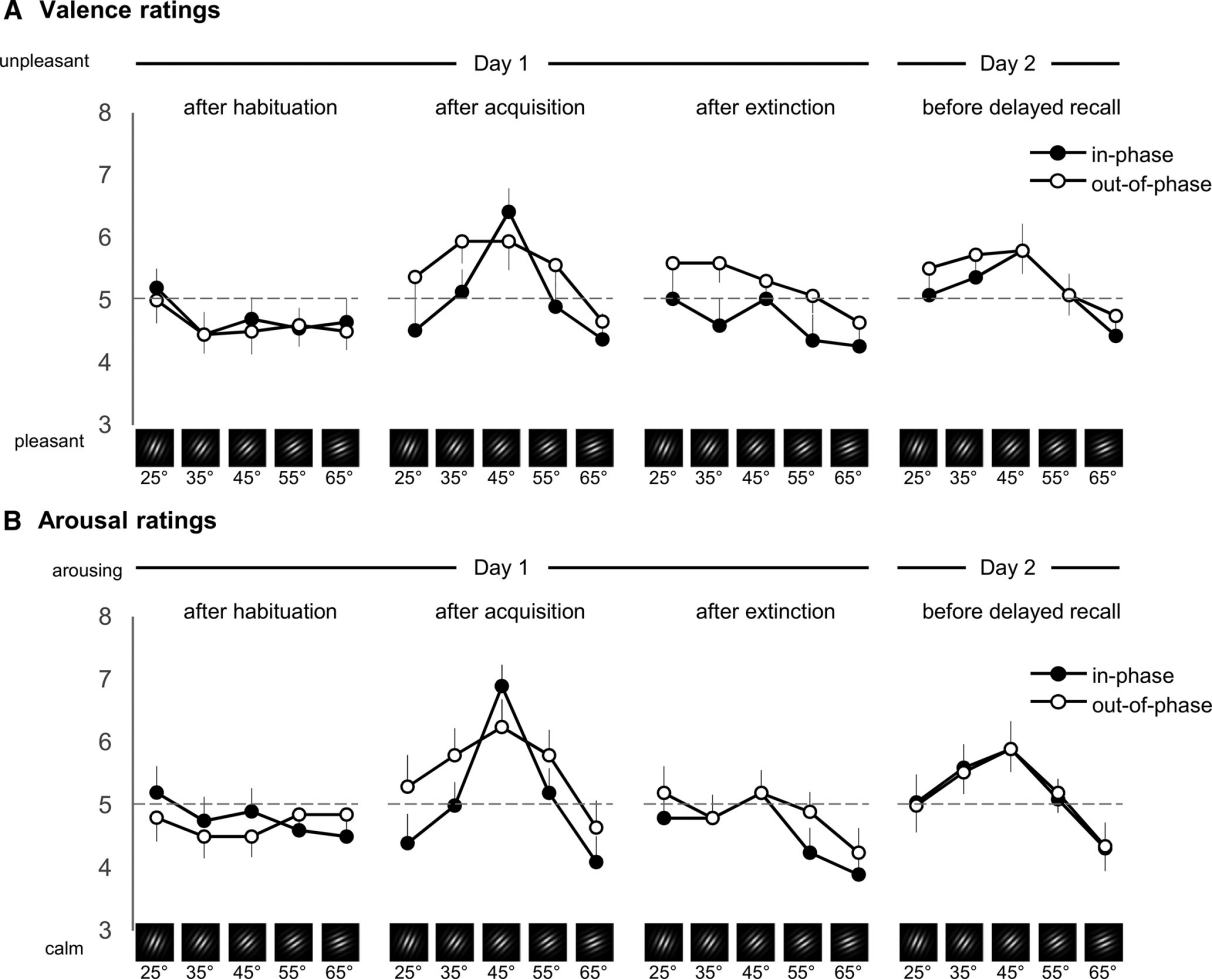
***A***, ***B***, Valence ratings (***A***) and arousal ratings (***B***) separated for each measurement point: after habituation, after acquisition, after extinction (day 1), and before delayed recall (day 2). Valence was rated with the Self-Assessment Manikin on a 9-point scale from 1 (unpleasant) to 9 (pleasant). For better comparability with arousal ratings, valence ratings were recoded, changing the scale from 1 (pleasant) to 9 (unpleasant). Arousal was also rated with the Self-Assessment Manikin, here ranging from 1 (calm) to 9 (arousing). Each data point presents valence or arousal ratings, respectively, for each CS orientation (averaged over participants per group and measurement point), error bars show 1 SEM. Note: for better visualization, the *y*-axis is scaled from 3 to 8 instead of showing the full range from 1 to 9. Extended Data Figure 6-1 shows discrimination indices (CS^+^ minus the weighted average of all CS^–^) and estimation statistics of valence and arousal data. Extended Data Figure 6-2 additionally shows the discrimination indices that use the unweighted average of all CS^–^ values for subtraction.

For both, valence and arousal ratings after acquisition (Fig. 6), we found a prioritization of the CS^+^ similar to that for US expectancy (main effect orientation: valence: *F*_(3,96)_ = 7.756; *p *=* *0.000272; partial η^2^ = 0.170; Table 1, l; arousal: *F*_(3100)_ = 10.928; *p *=* *0.000008; partial η^2^ = 0.223; Table 1, m). Again, reflecting the generalization around the CS^+^ (generalization fit: valence: *F*_(1,38)_ = 12.352; *p *=* *0.001; partial η^2^ = 0.245; Table 1, n; arousal: *F*_(1,38)_ = 19.587; *p *=* *0.000078; partial η^2^ = 0.340; Table 1, o). Here, mixed ANOVA showed no group main effect or orientation × group interaction for valence (Table 1, p, q) and arousal (Table 1, r, s). However, in both measures the in-phase group showed a narrower generalization than the out-of-phase group (Fig. 6). This was evident in significant orientation × group interactions in the form of a Mexican Hat for valence (*F*_(1,38)_ = 9.228; *p *=* *0.004; partial η^2^ = 0.195; Table 1, t) and arousal (*F*_(1,38)_ = 7.325; *p *=* *0.010; partial η^2^ = 0.162; Table 1, u). The discrimination indices (CS^+^ vs averaged CS^–^) as well as estimation plots, including individual values and effect sizes, are additionally presented in Extended Data Figure 6-1.

10.1523/ENEURO.0538-20.2021.f6-1 Figure 6-1 ***A***, ***B***, Weighted discrimination indices for valence ratings (***A***) and arousal ratings (***B***). Valence and arousal ratings were first *z*-transformed within each participant using the mean and SD of all ratings of valence and arousal of a participant, respectively. With the *z*-transformed data, we computed a weighted discrimination index per learning phase as the difference between the reinforced 45° (CS^+^) grating and the weighted average of the four CS^–^ gratings. Weights for the CS^–^ correspond to the angular difference in orientation between the four CS^–^ orientations (25°, 35°, 55°, 65°) and the CS^+^ orientation (45°): the two more similar CS^–^ orientations (±10° to the CS^+^) were weighted with 0.33[…], while the more dissimilar orientations (±20° to the CS^+^) were weighted with 0.166. Data and effect sizes are shown as a Cumming estimation plot (http://www.estimationstats.com). See the legend of Extended Data Figure 5-1 for a detailed description of a Cumming estimation plot. For valence data (***A***), the unpaired Hedge’s *g*: for habituation: –0.039 [95.0% CI, –0.680, 0.568], *p =* 0.896; for acquisition: –0.660 [95% CI, –1.219, 0.048], *p =* 0.0372; for extinction: –0.291 [95% CI, –0.925, 0.354], *p =* 0.3522; and delayed recall: –0.218 [95% CI, –0.832, 0.423], *p* = 0.4848. For arousal data (***B***), the unpaired Hedge’s *g*: for habituation: –0.296 [95% CI, –0.914, 0.386], *p =* 0.3372; for acquisition: –0.877 [95% CI, –1.459, 0.302], *p =* 0.0074; for extinction: –0.382 [95% CI, –1.020, 0.273], *p = *.2216, and for delayed recall, –0.142 [95% CI, –0.778, 0.510], *p =* 0.6472. The 5000 bootstrap samples were taken for CI estimation; the CI is bias corrected and accelerated. The two-sided *p* values are the likelihoods of observing the effect sizes, if the null hypothesis of zero difference is true. For each permutation *p* value, the 5000 reshuffles of the group labels were performed. Download Figure 6-1, TIF file.

10.1523/ENEURO.0538-20.2021.f6-2 Figure 6-2 ***A***, ***B***, Unweighted discrimination indices for valence (***A***) and arousal (***B***) ratings. Ratings were *z*-transformed within each participant using the mean and SD of all valence and arousal ratings of a participant, respectively. The unweighted discrimination index shown is the difference between ratings of the CS^+^ orientation and the unweighted average of the four CS^–^ orientations. Data and effect sizes are shown as a Cumming estimation plot (http://www.estimationstats.com). See the legend of Extended Data Figure 5-1 for a detailed description of a Cumming estimation plot. For valence data (***A***), the unpaired Hedge’s *g*: for habituation: 0.011 [95% CI, –0.622, 0.618], *p* = 0.9678; for acquisition, –0.578 [95% CI, –1.153, 0.047], *p* = 0.07; for extinction: –0.220 [95% CI, –0.864, 0.423], *p* = 0.488; for delayed recall: –0.218 [95% CI, –0.826, 0.422], *p* = 0.485. For arousal data (***B***), the unpaired Hedge’s *g*: for habituation: –0.255 [95% CI, –0.866, 0.439], *p* = 0.407; for acquisition: –0.820 [95% CI, –1.424, –0.225], *p* = 0.0128; for extinction: –0.361 [95% CI, –1.001, 0.295], *p* = 0.2466; for delayed recall: –0.141 [95% CI, –0.774, 0.503], *p *= 0.6512. The 5000 bootstrap samples were taken for CI estimation; the CI is bias corrected and accelerated. The two-sided *p* values are the likelihoods of observing the effect sizes, if the null hypothesis of zero difference is true. For each permutation *p* value, 5000 reshuffles of the group labels were performed. Download Figure 6-2, TIF file.

After extinction, there were no effects of synchronization in valence (group main effect or orientation × group interaction; Table 1, v, w) or arousal (group main effect or orientation × group interaction; Table 1, x, y). The same was true for valence and arousal ratings on day 2 (group main effect and orientation × group interaction: valence, Table 1, z, aa; arousal, Table 1, bb, cc).

### SCRs showed the typical increase toward the reinforced CS^+^ but were unaffected by the synchronization conditions

**Figure 7.**
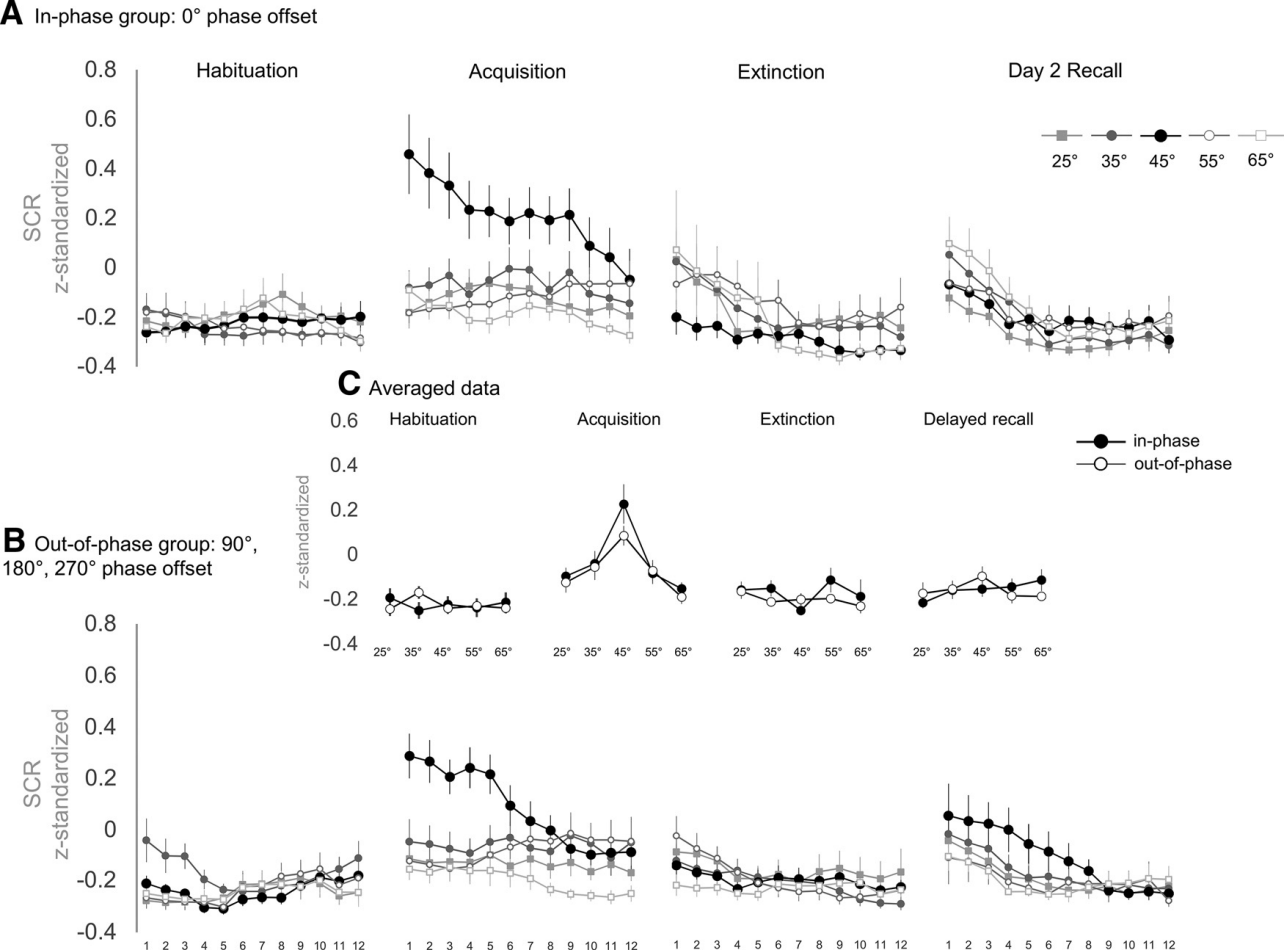
***A–C***, Single-trial (***A***, ***B***) and averaged (***C***) skin conductance responses. Single-trial SCRs are separated by the synchronization condition into the in-phase group (0° phase offset; ***A***) and the out-of-phase group (90°, 180°, and 270° phase offset; ***B***). Single-trial data are *z*-transformed SCRs, averaged over participants per group for each trial and CS orientation. Before averaging, data were smoothed over the 12 trials of a learning phase using a moving average (5 points long, symmetrical, shrinking at the end points). ***C*** depicts averaged data over 12 trials of habituation, acquisition, extinction, and delayed recall to visualize the response patterns within each learning phase. Here, each data point presents *z*-transformed SCRs of each CS orientation averaged over participants and trials per group. The *z*-transformation was calculated with the means and SDs over CS and US responses of all learning phases (habituation, acquisition, immediate extinction, delayed recall) per participant. Error bars show ±1 SEM. Extended Data Figure 7-1 shows single-trial SCR data without smoothing (i.e., no moving average). Extended Data Figure 7-2 shows discrimination indices (CS^+^ minus the weighted average of all CS^–^) for SCR and estimation statistics. Extended Data Figure 7-3 depicts discrimination indices without weighting the averaged CS^–^ values.

Figure 7, *A* and *B*, depicts the SCRs on a trial-by-trial basis to visualize the temporal dynamics of moving-averaged and *z*-transformed SCRs. In addition, *z*-values (i.e., without moving average) SCRs are presented in Extended Data Figure 7-1. However, as single trials are subject to noise, SCRs were analyzed using averaged data (Fig. 7*C*), as described in the Materials and Methods section. Pairing the CS^+^ orientation with the aversive US within acquisition led to the predicted increase of SCR toward the reinforced grating (main effect orientation: *F*_(3,96)_ = 14.856, *p *=* *3.1057E-7, partial η^2^ = 0.281; Table 1, dd). The response pattern was described by generalization around the CS^+^ (generalization fit: *F*_(1,38)_ = 31.987, *p* = 0.000002, partial η^2^ = 0.457; Table 1, ee). However, this was independent of group (main effect group and orientation × group interaction; Table 1, ff, gg, Extended Data Fig. 7-2 for discrimination indices and estimation statistics). Looking at Figure 7 (Extended Data Fig. 7-1), it is unusual that SCRs toward the CS^+^ seem already increased on the very first trial of acquisition, independent of the applied smoothing procedure (Extended Data Fig. 7-1, unsmoothed data). However, explorative analyses of group differences without the first trial did not change the results (i.e., there was still no overall difference between groups and no significant orientation × group interaction).

10.1523/ENEURO.0538-20.2021.f7-1 Figure 7-1 Single-trial data of SCRs without smoothing over trials. Same data as in Figure 7, *A* and *B*, plotted without the moving average over trials. SCRs are separated by learning phase (habituation, acquisition, extinction on day 1, and delayed recall on day 2) and by the synchronization condition into the in-phase group (i.e., 0° phase offset; ***A***) and the out-of-phase group (i.e., 90°, 180°, and 270° phase offset; ***B***). Error bars show ± 1 SEM. Download Figure 7-1, TIF file.

10.1523/ENEURO.0538-20.2021.f7-2 Figure 7-2 Weighted discrimination indices for averaged for averaged SCRs. SCRs were first *z*-transformed within each participant using the means and SDs over CS and US responses of all learning phases (habituation, acquisition, extinction, delayed recall). With the *z*-transformed data, we computed a weighted discrimination index per learning phase as the difference between the reinforced 45° (CS^+^) grating and the weighted average of the four CS^–^ gratings. Weights for the CS^–^ orientations correspond to the angular difference in orientation between the four CS^–^ orientations (25°, 35°, 55°, 65°) and the CS^+^ orientation (45°): the two more similar CS^–^ orientations (±10° to the CS^+^) were weighted with 0.33[…], while the more dissimilar orientations (±20° to the CS^+^) were weighted with 0.166[…]. Data and effect sizes are shown as a Cumming estimation plot (http://www.estimationstats.com). See Extended Data Figure 5-1 legend for a detailed plot description. The unpaired Hedge’s *g*: for habituation: –0.249 [95% CI, –0.827, 0.371], *p* = 0.451; for acquisition: –0.405 [95% CI, –0.938, 0.211], *p* = 0.2044; for extinction: 0.847 [95% CI, 0.277, 1.361], *p =* 0.0096; for delayed recall: 0.535 [95% CI, –0.091, 1.056], *p =* 0.0916. Download Figure 7-2, TIF file.

10.1523/ENEURO.0538-20.2021.f7-3 Figure 7-3 Unweighted discrimination indices for averaged SCRs. SCRs were *z*-transformed within each participant using the means and SD over CS and US responses of all learning phases (habituation, acquisition, extinction, delayed recall). The unweighted discrimination index shown is the difference between SCR to the CS^+^ and the unweighted average of the four CS^–^ orientations. Data and effect sizes are shown as a Cumming estimation plot (http://www.estimationstats.com). See Extended Data Figure 5-1 legend for a detailed plot description. The unpaired Hedge’s *g*: for habituation: –0.146 [95% CI, –0.754, 0.461], *p* = 0.6618; for acquisition: –0.385 [95% CI, –0.920, 0.230], *p* = 0.2296; for extinction: 0.754 [95% CI, 0.197, 1.259], *p* = 0.0212; for delayed recall: 0.549 [95% CI, –0.071, 1.059], *p *= 0.0848. The 5000 bootstrap samples were taken for CI estimation; the CI is bias corrected and accelerated. The two-sided *p* values are the likelihoods of observing the effect sizes if the null hypothesis of zero difference is true. For each permutation *p* value, 5000 reshuffles of the group labels were performed. Download Figure 7-3, TIF file.

During extinction, there was no difference between groups (main effect group and orientation × group interaction; Table 1, hh, ii) and also on day 2 synchronization had no effects (main effect group and orientation × group interaction; Table 1, jj, kk).

### ssVEP power revealed a tuning toward the visual CS^+^ that was similar in both groups

**Figure 8.**
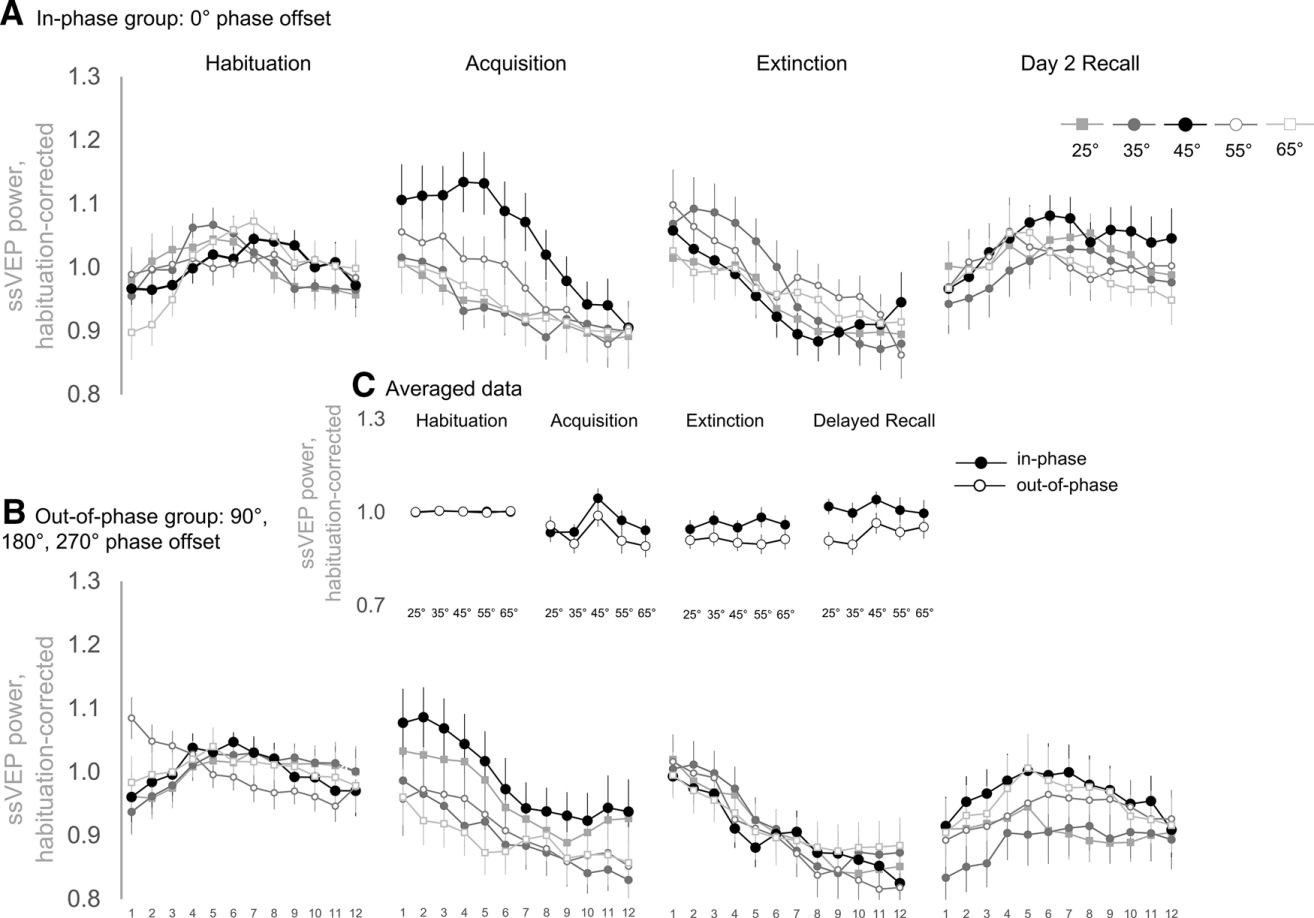
***A–C***, Single-trial (***A***, ***B***) and averaged (***C***) power of the 4 Hz ssVEPs for each learning phase (habituation, acquisition, extinction, and delayed recall). Single-trial data are separated by the synchronization condition into the in-phase group (0° phase offset; ***A***) and the out-of-phase group (90°, 180°, and 270° phase offset; ***B***). The ssVEP power is shown as the SNR at 4 Hz, corrected for habituation-level responding. Correction was performed by dividing individual SNR values by the average SNR from habituation (mean over all 60 trials of each participant, disregarding the different CS orientations). Therefore, values >1 describe an enhancement, and values <1 describe a decrease of ssVEP-SNR at 4 Hz relative to habituation. Single-trial data were smoothed over trials via a moving average along the 12 trials of each learning phase (5 point symmetrical shrinking at the end points). Each data point in ***A*** and ***B*** represents habituation corrected SNR for each trial and CS orientation, averaged over participants per group. ***C*** depicts data averaged over the 12 trials of habituation, acquisition, extinction, and delayed recall to visualize the response patterns within each phase. Error bars show ±1 SEM. Note: habituation data in ***C*** are nearly “flat” at ∼1 because of the habituation correction, as described above and in the Materials and Methods section. Extended Data Figure 8-1 shows single-trial data without the moving-average. Extended Data Figure 8-2 depicts discrimination indices with weighted CS- averages (CS+ minus weighted average of all CS-) and Extended Data Figure 8-3 shows discrimination indices without weighting the averaged CS- responses.

Figure 8, *A* and *B*, depicts ssVEPs on trial-by-trial basis to visualize temporal dynamics, and Extended Data Figure 8-1 shows ssVEP ratios without a moving average. However, as for the SCRs, ssVEPs were analyzed using averaged data (Fig. 8*C*), as described in the Materials and Methods section.

10.1523/ENEURO.0538-20.2021.f8-1 Figure 8-1 Single-trial power of the 4 Hz ssVEPs without smoothing over trials. Same data as in Figure 8, *A* and *B*, plotted without the moving average over trials. Single trials are separated by learning phase (habituation, acquisition, extinction on day 1, and delayed recall on day 2) and by the synchronization condition into the in-phase group (i.e., 0° phase offset; ***A***) and the out-of-phase group (i.e., 90°, 180°, 270° phase offset; ***B***). Error bars show ±1 SEM. Download Figure 8-1, TIF file.

10.1523/ENEURO.0538-20.2021.f8-2 Figure 8-2 Weighted discrimination indices for ssVEPs. Within each learning phase, using the habituation corrected SNR at 4 Hz (Fig. 8*C*), we computed a weighted discrimination index per learning phase as the difference between the reinforced 45° (CS^+^) grating and the weighted average of the four CS^–^ gratings. Weights for the CS^–^ correspond to the angular difference in orientation among the four CS^–^ orientations (25°, 35°, 55°, 65°) and the CS^+^ orientation (45°): the two more similar CS^–^ orientation (±10° to the CS^+^) were weighted with 0.33[…], while the more dissimilar orientations (±20° to the CS^+^) were weighted with 0.166[…]. Data and effect sizes are shown as a Cumming estimation plot (http://www.estimationstats.com). See Extended Data Figure 5-1 legend for a detailed plot description. The unpaired Hedge’s *g*: for habituation: 0.008 [95% CI, –0.652, 0.633], *p* = 0.979; for acquisition: –0.114 [95% CI, –0.731, 0.511], *p *= 0.7084; for extinction: 0.130 [95% CI, –0.519, 0.741], *p *= 0.683; for delayed recall: 0.054 [95% CI, –0.564, 0.702], *p *= 0.08622. The 5000 bootstrap samples were taken for CI estimation; the CI is bias corrected and accelerated. The two-sided *p* values are the likelihoods of observing the effect sizes if the null hypothesis of zero difference is true. For each permutation *p* value, 5000 reshuffles of the group labels were performed. Download Figure 8-2, TIF file.

10.1523/ENEURO.0538-20.2021.f8-3 Figure 8-3 Unweighted discrimination indices for ssVEPs. Here, the discrimination index was computed as the difference between the reinforced 45° orientation (CS^+^) grating and the unweighted average of the four CS^–^ orientations. Data and effect sizes are shown as a Cumming estimation plot (http://www.estimationstats.com). See Extended Data Figure 5-1 legend for a detailed plot description. The unpaired Hedge’s *g*: for habituation: –0.074 [95% CI, –0.708, 0.569], *p* = 0.8106; for acquisition: –0.161 [95% CI, –0.774, 0.464], *p* = 0.6074; for extinction: 0.080 [95% CI, –0.561, 0.706], *p* = 0.7948; for delayed recall: 0.044 [95% CI, –0.579, 0.687], *p* = 0.891. The 5000 bootstrap samples were taken for CI estimation; the CI is bias corrected and accelerated. The two-sided *p* values are the likelihoods of observing the effect sizes, if the null hypothesis of zero difference is true. For each permutation *p* value, 5000 reshuffles of the group labels were performed. Download Figure 8-3, TIF file.

ssVEPs during acquisition revealed a conditioned power increase toward the CS^+^ and neighboring gratings (main effect orientation: *F*_(4,137)_ = 5.696, *p *=* *0.000479, partial η^2^ = 0.130; Table 1, ll). It was described by a generalization pattern around the CS^+^ (generalization fit: *F*_(1,38)_ = 8.447, *p *=* *0.006, partial η^2^ = 0.182; Table 1, mm). However, this prioritization was not affected by group (orientation × group interaction; Table 1, nn). In similarity to SCRs during acquisition, Figure 8, *A* and *B*, indicates an increased ssVEP ratio toward the CS^+^ on the very first trial. However, considering the unsmoothed data in Extended Data Figure 8-1, the power increase here seems to be an artifact of the applied smoothing procedure. As depicted in Figure 8*C*, synchronization also had no effects on ssVEPs in extinction. Consequently, we found neither a significant main effect of group nor an orientation × group interaction (Table 1, oo, pp). Intriguingly, the ssVEP power during delayed recall on day 2 was generally higher in the in-phase group than in the out-of-phase group (*F*_(1,38)_ = 5.354, *p *=* *0.026, partial η^2^ = 0.123, Table 1, qq), although this effect was independent of orientation (orientation × group interaction; Fig. 8*C*, Table 1, rr). In accordance with ratings and the SCRs, discrimination indices (weighted CS^+^ minus averaged CS^–^ gratings) and estimation plots depicting individual values and effects sizes are presented in Extended Data Figure 8-2.

## Discussion

The formation of associative memories is an elemental aspect of human behavior, but its underlying neurocomputations are largely unknown. One group of theoretical notions has emphasized the role of phase-synchronized oscillations for binding representations of conditioned cues to behavioral outcomes (Headley and Paré, 2017). Recent research has increasingly used external rhythmic stimulation to test the role of phase relations in specific frequency bands for the formation of working memory (Polanía et al., 2012; Violante et al., 2017) and audiovisual associations (Clouter et al., 2017; Wang et al., 2018). Based on these previous findings, we applied this method for the first time in a fear-conditioning paradigm. Modulating the phase shift of a visual CS and aversive auditory US that was presented in the memory-relevant theta frequency allowed us to causally interpret phase synchronization in fear conditioning. To assess the various response systems that are important in fear learning (Lang et al., 2000), we measured skin conductance responses, indexing the physiological arousal of fear; the ratings of valence and arousal to capture the subjective evaluation of each stimulus; and US expectancy, which assesses the participant’s knowledge of the CS–US association. Additionally, ssVEPs provided information about visuocortical engagement and tuning patterns in sensory processing.

The measures we collected in the current study reflect different facets of the associative conditioning process and as such responded differently to the experimental manipulations. In accordance with our hypothesis, synchronized CS–US presentation facilitated the ability to identify the CS^+^ as the grating that was most likely followed by the aversive US. Remarkably, participants that received synchronized CS–US presentation discriminated the CS^+^ more precisely from the neighboring CS^–^ gratings (which only differed in an orientation shift of 10°). Participants in the out-of-phase group, in contrast, generalized across the CS^+^ or the most similar CS^–^ gratings. We therefore conclude that the synchronous input of two (multimodal) stimuli stemming from two sensory modalities strengthens the cognitive representation of the CS–US association.

Consistent with the US expectancies, the subjective valence and arousal ratings reflected the effects of phase synchronization. While participants who received in-phase stimulation were more sensitive to changes in the perceived valence and arousal of the CS^+^, participants in the out-of-phase group reported generalized arousal and unpleasantness across the CS^+^ and neighboring CS^–^ gratings. Hence, synchronous input not only sharpens the cognitive representation of CS–US contingency but seems to have a similar influence on the affective evaluation.

Surprisingly, there were no corresponding effects in the SCR or ssVEP data. Considering SCR data, during acquisition both groups showed the strongest response toward the CS^+^ grating, independent of synchronization. Especially in the single-trial data, however, the in-phase group appears to respond stronger to the CS^+^, which seems to be more pronounced in the first trials. We therefore exploratively tested the potential group difference by segmenting the trials into trial blocks (three blocks with four trials per block). However, adding this within factor to our statistical analysis did not reveal any significant differences between the groups in different phases of acquisition. One possible explanation of the higher SCRs in the beginning of acquisition might be the booster sequence that was used (i.e., five of the first seven gratings were CS^+^ gratings). The booster and the applied criterion to not allow more than two consecutive CS of the same orientation might also be the reason for another unusual observation within our SCR results: irrespective of the factor group and independent of the applied smoothing procedure, SCR toward the CS^+^ was already increased on the very first trial. While we applied the booster sequence for a better comparability with previous findings (McTeague et al., 2015; Antov et al., 2020), future studies should consider a different approach to minimize the similar trial order effect. Nevertheless, there were no differences between the in-phase and out-of-phase group; thus, this observation does not change the following interpretations.

An effect of phase synchronization was also missing in the ssVEP-data. Although we were able to detect a tuning pattern with the greatest power for the reinforced CS^+^ grating for 4 Hz stimulus presentation as previously described for stimulation in the low beta range (McTeague et al., 2015; Antov et al., 2020), the pattern did not differ between in-phase and out-of-phase group.

A possible explanation for the observed discrepancies in the different variables could be the involvement of different memory types we might have assessed with our measures. Although fear conditioning is a well established and widely used paradigm, it is difficult to strictly distinguish the mechanisms behind each response system. For example, skin conductance responses measure physiological arousal during fear learning (although it is not restricted to fear conditioning) and is often considered as a measure of the unaware fear reaction, especially dependent on the amygdala (Knight et al., 2003, 2006; Christopoulos et al., 2019; but see also Lovibond and Shanks, 2002; Sevenster et al., 2014). US expectancy ratings, on the contrary, are considered to specifically reflect declarative knowledge of the CS–US contingency (Boddez et al., 2013), which is known to include additional brain structures like the hippocampus. Bechara et al. (1995) observed a neural dissociation between implicit and explicit aspects of a fear-conditioning procedure. While patients with bilateral amygdala lesions were unable to elicit SCRs but had an intact memory for the declarative facts, patients with bilateral lesions of the hippocampus showed the exact opposite effects (i.e., they acquired SCRs but failed to recall declarative facts). Speculating that the effects of visual–auditory stimulation is not only restricted to early sensory cortices, but influences deeper brain regions in the course of rhythmic processing, our results might be explainable based on these distinct systems: theta phase synchronization might especially modulate the path involved in forming declarative facts about the CS–US pairing (i.e., US expectancy ratings), probably including the hippocampus, without influencing the emotional conditioning comprising the amygdala. One possible mechanism could be that the phase-synchronous visual CS^+^ and auditory US simultaneously arrive at neural populations in the hippocampus, increasing the likelihood of long-term potentiation and thereby enhancing synaptic strength (Fell and Axmacher, 2011). Although the EEG method used here does not allow the drawing of conclusions about mechanisms at the synaptic level in subcortical structures, one might also speculate why the heightened CS–US association in the rating data is not reflected in metrics thought to reflect limbic processing (i.e., the SCRs). There are two potential explanations that we highlight in this context. (1) In various species, theta-phase synchronization has predominantly been examined in the context of LTP in the hippocampus (Huerta and Lisman, 1995; Buzsáki, 2002; Hyman et al., 2003; Lega et al., 2012), which is specifically relevant in the formation of declarative memory (Eichenbaum, 1999; Clouter et al., 2017; Wang et al., 2018). Thus, one may speculate that theta-phase synchronization is linked to hippocampus-dependent processes, whereas the exact timing of CS and US may play a lesser role in amygdala-dependent fear learning. However, some studies have found theta-phase synchronization between the amygdala and other important structures of the fear circuit (e.g., hippocampus, ventrolateral PFC, anterior gyrus cinguli) as well as within the subnuclei of the amygdala (Seidenbecher et al., 2003; Karalis et al., 2016; Taub et al., 2018; Zheng et al., 2019; for review, see Bocchio et al., 2017; Çalişkan and Stork, 2018) during different stages of the fear-conditioning process, supporting the general influence of theta synchronization during fear memory formation. As such, future work may characterize the role of synchronization within and between specific brain regions for the establishing and maintenance of fear memories. (2) More importantly, however, is the question whether synchronized theta rhythms propagate to the amygdala. Sensory information reaches the amygdala via multiple pathways, among which some are faster and subcortical, or “low,” routes; and others are slower, or “high,” cortical routes (Silverstein and Ingvar, 2015). Since our method of visual and auditory synchronized (vs asynchronized) theta stimulation is delivered globally and is unlikely to target one specific pathway, the timing might not have been suitable to enable locally specific synchronization. Considering that we used a generalization paradigm with similar CS gratings, we may offer the speculation that the challenging discrimination of the CS^+^ requires a more demanding processing via the slow, cortical route, while the simple aversive US reaches the amygdala via the fast, subcortical pathway. Thus, the 40 ms we added to the US might have been insufficient to achieve theta synchronization when the CS and US reach the LA. Because of the relatively long CS–US overlap of 2 s, we additionally cannot rule out that our synchronized stimulation reached the amygdala via the thalamic route first, but then also via cortical routes, leading to cancellation of the first CS–US phase synchronization, hence minimizing the suggested effects.

Another interesting consideration in this context is the role of theta synchronization between the amygdala and hippocampus for pattern separation of emotional images. Examining presurgical epilepsy patients, Zheng et al. (2019) found that bidirectional theta synchronization between both structures was associated with the ability to discriminate an encoded image and a new, but similar, “lure” image in a test phase. Considering that most of our results consist of a better discrimination between the aversive CS^+^ and the most similar CS^–^ gratings, synchronized CS–US presentation might be beneficial for the amygdalo–hippocampal communication, associated with enhanced discrimination of emotional content. However, further research with additional outcome measures is needed to pinpoint all underlying neurophysiological processes. For example, future studies may attempt to experimentally untangle declarative and nondeclarative memory processes involved in fear conditioning, including their reactivity to synchronized presentation. Measuring amygdala and hippocampus activity via fMRI or in experimental animals may also help to clarify the influence of synchronized presentation on distinct subprocesses of fear conditioning and their associated neural substrates.

One important consideration when interpreting the current results is the fact that group differences were mostly restricted to the encoding phase of fear (acquisition), although we expected that improved fear learning after synchronous presentation prompts greater extinction resistance. Contrary to expectations, we did not find extinction-resistant patterns in the in-phase group during immediate extinction or delayed recall. However, using a reinforcement rate of 100% is known to cause rapid extinction (Haselgrove et al., 2004; Dunsmoor et al., 2007), which could make it harder to detect between-group effects. Moreover, because extinction leads to the formation of a new (i.e., CS–no-US) memory trace that inhibits the original fear memory, future research may use a second CS^+^ stimulus that is not extinguished, which will aid in assessing the long-lasting effects of theta-phase synchronization on fear memory recall (i.e., the trace that was causally manipulated by theta-synchronized stimulation). Additionally, animal and human work suggests that prolonged stimulus-free periods during encoding are associated with the more effective production of long-term memory (Philips et al., 2013; Jiang et al., 2020). Increasing the ITIs might help to form more robust memory traces that persist over time. Another possible cause for the absence of long-term effects of stimulation phase is that the externally modulated CS–US stimulation only affects short-term or working memory processes but does not have any effects on actual long-term memory. In two comparable studies focusing on declarative memory Clouter et al. (2017) and their follow-up study by Wang et al. (2018) used a distractor task as a time gap between the encoding and recall of the learned video–tone associations, which only lasted for 30 s, likely too short a time to inform the formation of long-term memory.

Although the current study provided evidence of a causal role of theta-phase synchronization in the context of fear conditioning, there are some limitations to consider. First, our sample size was chosen to detect medium to strong effect sizes, which was based on previous studies (Clouter et al., 2017; Wang et al., 2018). Therefore, we cannot rule out the possibility that we could not detect small effect sizes. This is especially interesting for the SCR data, where the responses are descriptively stronger after in-phase CS–US presentation, but the statistics did not show significant differences. Increasing the statistical power via a greater sample size might help to even detect small effect sizes. Second, we cannot conclude that the stimulation effects observed here are specific to the theta band, because we did not test other frequencies. However, both animal model studies that examined theta-phase synchronization in the fear network (Seidenbecher et al., 2003; Taub et al., 2018), as well as entrainment studies that focused on working and declarative memory (Alekseichuk et al., 2016; Clouter et al., 2017; Violante et al., 2017) support the current conclusion that synchronization in the theta-frequency band is specifically important for fear memory formation. Third, we did not explicitly ask whether participants were able to detect the synchronous or asynchronous timing between CS and US, and therefore we cannot rule out that out-of-phase or in-phase stimulation exerted effects based on phenomenological, perceptual differences. Nevertheless, we used the exact time lags used by both Clouter et al. (2017) and Wang et al. (2018), who did not observe any interference with perceptual judgments or decline in performance. In addition, Clouter et al. (2017) conducted a control experiment with static stimuli, which, on a perceptual basis, represents the best-case scenario for perceptual binding and still found better results after theta-synchronized video–audio presentation. Fourth, although EEG data showed a group-independent tuning toward the CS^+^ grating, supporting the conditioned effects on sensory processing, the hypothesized sharpening in the in-phase group was not confirmed. What we found is a general increase in ssVEP-power in the in-phase group during day 2, suggesting a stronger engagement of the sensory cortex. However, based on the present data, we cannot establish to what extent this effect was caused by the theta-phase synchronization on day 1 as opposed to arising as an epiphenomenon (e.g., of the cognitive changes induced by the synchronization). Fifth, theta synchronization may not facilitate learning, but desynchronized stimulation may disrupt ongoing oscillatory processes, resulting in less precise (i.e., more generalized) fear responses (Alekseichuk et al., 2017). To clarify this assumption, future work may include a third group in which participants are presented with nonflickering CS and US stimuli.

Finally, an important limitation is that we were not able to show that participants’ auditory and visual EEG responses were synchronized or desynchronized as intended. This was because of the limited number of trials and noisy US data. In contrast to previous work with innocuous stimuli, the US in a fear-conditioning experiment has to be highly aversive. Inherently, this means that the duration of the US (the only period in which auditory and visual stimulation overlap) will produce noisy EEG data with many movements, a startle response, and other artifacts. This is why EEG studies of human fear conditioning (regardless of the US used and the number of trials) typically do not analyze any data during the US presentation window. Because the US is aversive, we did not want to expose participants to more noise than absolutely necessary. Thus, based on previous experiments, we limited the duration of a single US to 2000 ms and the number of US trials to 12 per participant. In combination with inherently noisy EEG data during a US presentation precluded us from localizing and analyzing phase differences in the brain response. This should be addressed with a modified design in future studies. Nevertheless, we did verify the precise nature of the bimodal stimulation on a single-trial and single-subject level (Fig. 2). Importantly, earlier work (Clouter et al., 2017; Wang et al., 2018) with 4 Hz audio–video synchronization using larger trial numbers and nonaversive audio stimulation have shown that precise audio–video stimulation results in synchronized responding in the auditory and visual cortex. Future studies may also consider extending the temporal gap between the acquisition phase and the delayed recall, because previous work has indicated that theta-band synchronization between the amygdala and sensory cortices affects the storage of fear information in remote, but not recent, fear retrieval (Sacco and Sacchetti, 2010; Do-Monte et al., 2015).

In conclusion, the current study represents an initial step toward establishing the causal effects of theta-phase synchronization for fear memory formation. Our results replicate the importance of synchronization for acquiring new cognitive representations, measured via US expectancy ratings, and affective evaluation (subjective valence and arousal ratings). By contrast, the present evidence was mixed at the level of sympathetic (skin conductance) and visuocortical (ssVEPs) engagement. Future studies may further explore the differentiation between different response systems in the context of fear conditioning. Leveraging the potential of rhythmic stimulation and synchronization while taking into account the evolution of fear acquisition across the learning phases will ultimately assist in improving our understanding of the mechanisms behind the acquisition of learned fear responses.

## Synthesis

Reviewing Editor: Satu Palva, University of Helsinki Decisions are customarily a result of the Reviewing Editor and the peer reviewers coming together and discussing their recommendations until a consensus is reached. When revisions are invited, a fact-based synthesis statement explaining their decision and outlining what is needed to prepare a revision will be listed below. The following reviewer(s) agreed to reveal their identity: NONE.

This study demonstrates an interesting new approach to study the effect of synchronous oscillations on cognition. More specifically, the study demonstrates a causal role of 4Hz synchronization to fear conditioning. While this has been demonstrated for multisensory episodic memories, such a role has not been shown in fear conditioning before. The authors used external rhythmic sensory stimulation at 4 Hz to investigate if fear conditioning is influenced by synchronously presented conditioned stimulus (CS) and unconditioned stimulus (US) as compared to asynchronously presented CS and US, which were both modulated at 4 Hz. They showed that participants’ subjective ratings on the expectation of an upcoming US, valence and arousal were more precise in the in-phase group than the out-of-phase group, which the ratings of the out-of-phase group showed broader generalised pattern from CS+ orientation to the adjacent orientations. Physiological results, as measured by Skin Conductance Responses (SCR) and Steady-state visual evoked potentials (ssVEP) did not show similar findings as the subjective ratings, but showed a general stronger tuning by CS+, regardless of phase group. The findings are interesting and have translational impact that might help to develop economical and efficient tool to help treat mental disorders such as Post-traumatic stress disorder (PTSD).

Major

1. The authors employed a recently developed paradigm to manipulate the degree of theta phase synchronisation, which the original study by Clouter et al. (2017) had an elegant hypothesis on the causal role of theta phase synchronisation in associative memory, given the well-established animal literature on hippocampal theta phase supporting long-term potentiation (LTP) and long-term depression (LTD). However, it is not clear enough how and why the authors expected that the synchronised presentation of CS and US at 4 Hz would promote better association of CS and US. The authors reviewed literature on theta oscillations and fear network involving the amygdala, hippocampus, and medial prefrontal cortex (mPFC). It would be better if the authors can elaborate on the possible mechanisms that enhance encoding of associations. For example, why the author’s would expect a difference in CS and US association if the stimulus input timing difference is just 65 - 125 ms.

2. The authors discussed a possible mechanism of why SCR did not show any group effect like it showed in the subjective ratings was that subjective ratings reflect declarative memory, which is processed in the hippocampus while SCR depends on the amygdala. The question is this: Did the authors have any hypothesis that the theta phase synchronisation would mediate the amygdala activity or the hippocampal-amygdala circuit? Addressing these issues would make the storyline clearer.

3. Neuronal synchronization should be better explained in the beginning of the Introduction. Also frequencies other than that between 4-8 Hz should be considered and it should be made clear what is meant by the theta oscillation. Where is it assumed to originate, for example? Also please make sure to mention the species in which a specific finding has been made as theta-band oscillations recorded in the mouse brain are not the same thing as 4-8 Hz oscillations detected in human MEG recordings, for example.

4. Please report the exact nature of effects/connections between factors instead of just referring to “a connection”. Example: “Weather, death by drowning and ice cream consumption are linked. -> Both ice cream consumption and death by drowning is more common during periods of hot weather compared to cold weather.”

5. Five visual Gabor gratings of orientations at 25, 35, 45, 55 and 65 served as CS, with only the 45 degrees orientation as reinforced CS+. Why didn’t the authors counterbalance the reinforced CS+ for each orientation across participants? Can the authors justify this?

6. In Page 7, ‘The luminance of the visual CS was sinusoidally modulated in 4 Hz, where luminance changed at each screen refresh, resulting in 21 steps per half cycle...’, according to Figure 1C and 85 Hz refresh rate of the monitor, should here be ‘per cycle’ rather than ‘per half cycle’?

7. In Page 8, Figure 1C, In-phase group, both legends are ‘4-Hz modulated luminance of visual CS’, should the lower one be ‘4-Hz modulated volume of auditory CS’?

8. In Page 9, ’Conditioning procedure’ session, the length of CS+ was 2 s longer than the length of CS. Thus, I would be cautious to interpret any effect of main effect of orientation only, without any interaction, as this could be caused purely by longer trial presentation time in the CS+.

9. The authors used a different ground for the EOG, is there any specific reason for doing that?

10. In Page 15, Figure 3, authors did CSD and projected back to the scalp and plotted the distribution of the SNR. Do the topos look typical for ssVEP and ASSR? Especially for the ssVEP, why there are two separate clusters over parietal and occipital areas?

11. In Page 15, the ’ssVEP single-trial analysis’ section, the authors up sampled the data to 1536 Hz, can the authors justify the necessity of doing this?

12. In Page 18, Overall Procedure, can the authors state when the screening session happened? Did the screening session happen on the same day as the main conditioning session?

13. It is not clear what task participants were asked to do during the 24-h delayed recall, can the authors give details about this?

14. In Page 19, the ’Statistical analysis’ session, the first sentence, ’We subjected...’, do the authors meant ’submitted’?

15. In Page 20, Figure 4B, it seems to me that you can only get a resulting shape like this if you subtract the broad generalization from the narrow generalization, not the other way around, can the authors plot the model weights for the narrow generalization as well?

16. In Page 24, Figure 5, the data for Day 2 before delayed recall still showed an interesting pattern that there was more precise tuning (highest rating in 45) for the in-phase group, while a non-CS+ orientation showed the highest rating for the out-of-phase group. Could the authors statistically test this, e.g. do an ANOVA of group (in-phase vs. out-of-phase) and orientation (45 vs. the highest rating of the other four orientations) and see if there is an interaction?

17. In Page 25, Figure 6, this figure might look more intuitive if the authors change the Valence ratings y-axis from pleasant (low) to unpleasant (high).

18. In Page 27, Figure 7A, there seems to have an effect on trial order in the Acquisition, Extinction and Day 2 Recall phases. The first several trials showed stronger SCR than the later trials and this looks more robust in the in-phase group than the out-of-phase group. Have the authors considered to quantify this effect?

19. In Page 27, Figure 7C, it looks to me as if the in-phase group was more precise in tuning to the CS+ than the out-of-phase group. Can the authors do a simple ANOVA of group (in-phase vs. out-of-phase) and orientation (45 vs. the mean of the other four orientations)?

20. In Page 27, ssVEP session, the authors showed topos of both visual and auditory SSEP in the Methods section. I would expect that the authors do the same analysis as done in Clouter et al., 2017, i.e. extract time series of conditioning from visual and auditory regions that are pre-defined by the unimodal topos for each group and see if the phase offset between the two time series are as manipulated, e.g. 0 or 90, 180, 270, which could show that the manipulation was valid in the brain.

21. In Page 29, Figure 8A and B, the ssVEP was analysed during the 5 s flicker presentation but in the Acquisition phase, the ssVEP for 45 in both groups started to be larger than the other orientations since the very first trial, can the authors explain why? Was this statistically significant? Was the sequence somehow predictable?

22. At the end of Page 33 and the beginning of Page 34, the authors discussed about whether the main effect of group found for ssVEP in day 2 was caused by the theta phase synchronisation on day 1. Can the authors do the same analysis as done in Clouter et al. 2017 to compute phase differences between time series extracted from visual ROI and auditory ROI for each participant and get a resultant vector length value for each participant to correlate with the day 2 ssVEP to see if there was a positive correlation between the resultant vector length value in day 1 and ssVEP in day2 for the in-phase group, and if there was a negative correlation between the value in day 1 and ssVEP in day 2 for the out-of-phase group. If the day 2 ssVEP main effect of group reflects better association by theta phase synchronisation in day 1, then the better a participant from the in-phase group was entrained in day 1, the stronger the ssVEP in day 2 should be. In contrast, if a participant was entrained well to the out-of-phase stimulation, then they should have weaker ssVEP in day 2.

23. In the beginning of Page 33, the authors discussed about a control condition of non-flickering CS and US, I wonder if there is any existing literature using similar paradigm that the authors could compare their findings to?

24. On page 3, paragraph ends in “Thus, if synchronized, i.e., temporally orchestrated, neuronal activity heightens inter-area communication and plasticity, the synchronized input into the two sensory systems processing the CS and the US should strengthen the formation of a CS-US association. “ Divide this into two sentences. Also, as such, the preceding text does not really explain the mechanisms of why it should be like this. That is, this conclusion is not very convincingly justified.

25. Is there a reason for using a very short inter-trial interval (3- to 5 s) compared to the duration of the actual stimulus presentation (up to 7 s)? This could have an effect on learning and in future studies it might be beneficial to allow a considerably longer stimulus-free period between the trials. See for example: https://www.frontiersin.org/articles/10.3389/fnmol.2019.00317/full

26. On page 20 it says: “We obtained the contrast weights by subtracting a broader generalization profile from a narrower generalization profile (Figure 4 B)”. However, the figure shows the opposite, that is, the narrower curve being subtracted from the broader curve. Which is the way you did it?

Regarding Table 1 stating the statistics, please include in the note/table caption some information about the meaning of the lines a to rr. Now it is impossible to interpret the table alone, without reading the results section text.

27. On page 32 the authors mention the effect of theta on hippocampus and LTP. Perhaps add the following (or other similar) papers as references and discuss their findings:

https://pubmed.ncbi.nlm.nih.gov/25979993/

https://pubmed.ncbi.nlm.nih.gov/14684874/

28. Could the short inter-stimulus interval have something to do with the effects being limited to Day 1 Acquisition session?
