# Supplementary figures and images for: Phase-Synchronized Stimulus Presentation Augments Contingency Knowledge and Affective Evaluation in a Fear-Conditioning Task
Source: eNeuro. 2022 Jan 5;9(1):ENEURO.0538-20.2021. doi: 10.1523/ENEURO.0538-20.2021 (PMC8751852; doi:10.1523/ENEURO.0538-20.2021)

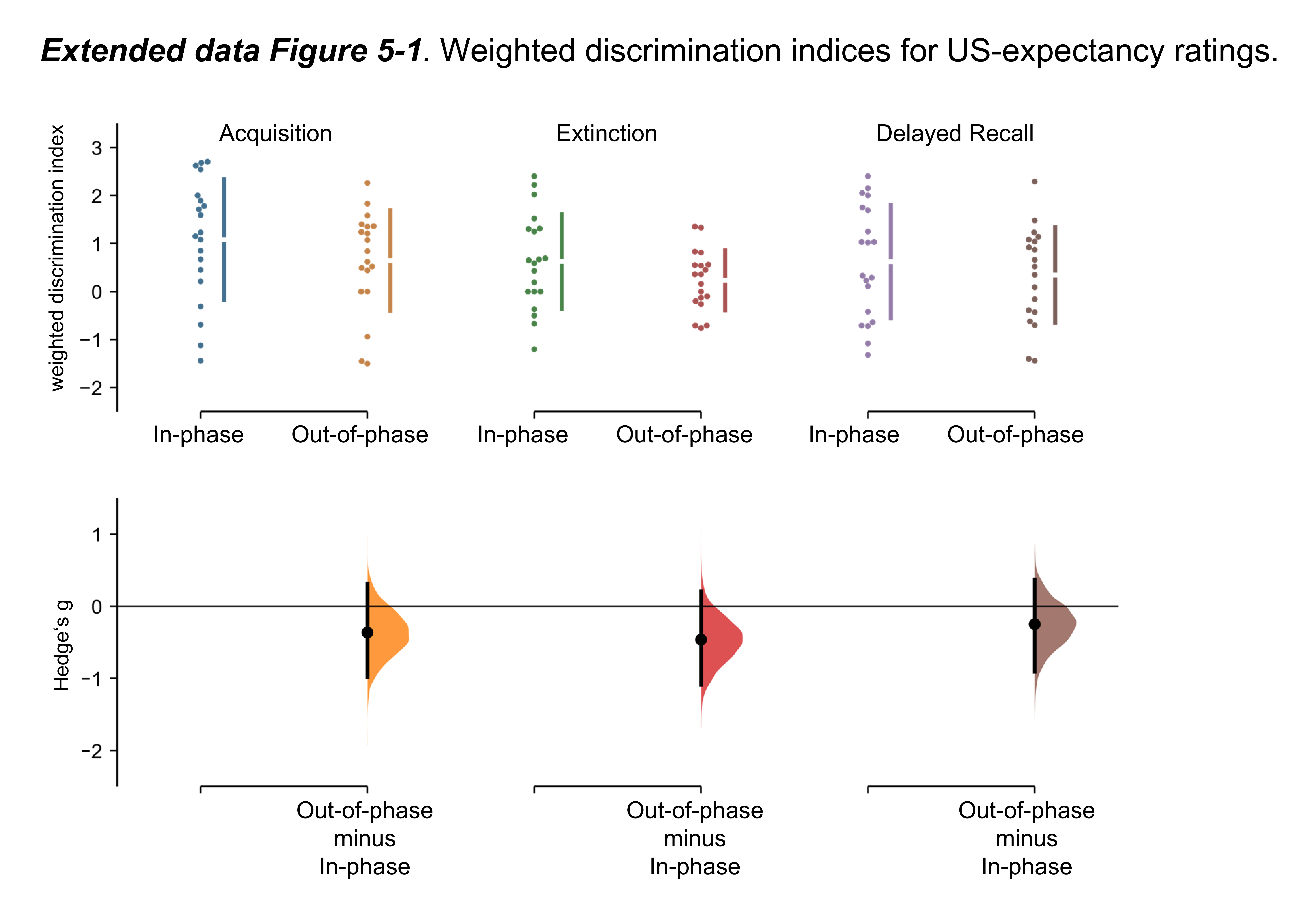

Supplement: Figure 5-1 — Weighted discrimination indices for US expectancy ratings. US expectancy ratings were first z-transformed within each participant using the mean and SD of all US expectancy ratings of a participant. With the z-transformed data we computed a weighted discrimination index per learning phase as the difference between the rating of the reinforced 45° (CS+) grating and the weighted average of the four CS– gratings. Weights for the CS– correspond to the angular difference in orientation between the four CS– orientations (25°, 35°, 55°, 65°) and the CS+ orientation (45°). The two more similar CS– orientations (±10° to the CS+) were weighted with 0.33[…], while the more dissimilar orientations (±20° to the CS+) were weighted with 0.166[…]. Data and effect sizes are shown as a Cumming estimation plot (http://www.estimationstats.com). Top row, Swarm plots show the raw discrimination indices per learning phase (each dot is the discrimination index of one participant). Group statistics are indicated to the right of each swarm as gapped lines (gap = mean, line length = 1 SD). Bottom row, Effect size estimates (Hedges’ g, black dots) for the three relevant comparisons (in-phase vs out-of-phase for each learning phase) and their 95% confidence interval (CI; vertical error bars). The unpaired Hedge’s g: for acquisition: –0.364 [95% CI, –0.981, 0.315], p = 0.2578; for extinction: –0.463 [95% CI, –1.089, 0.205], p = 0.1532; for delayed recall: –0.249 [95% CI, –0.907, 0.370], p = 0.4206. The 5000 bootstrap samples were taken for CI estimation; the CI is bias corrected and accelerated. The two-sided p values are the likelihoods of observing the effect sizes, if the null hypothesis of zero difference is true. For each permutation p value, 5000 reshuffles of the group labels were performed. Download Figure 5-1, TIF file. [file enu-eN-NWR-0538-20-s03.tif]

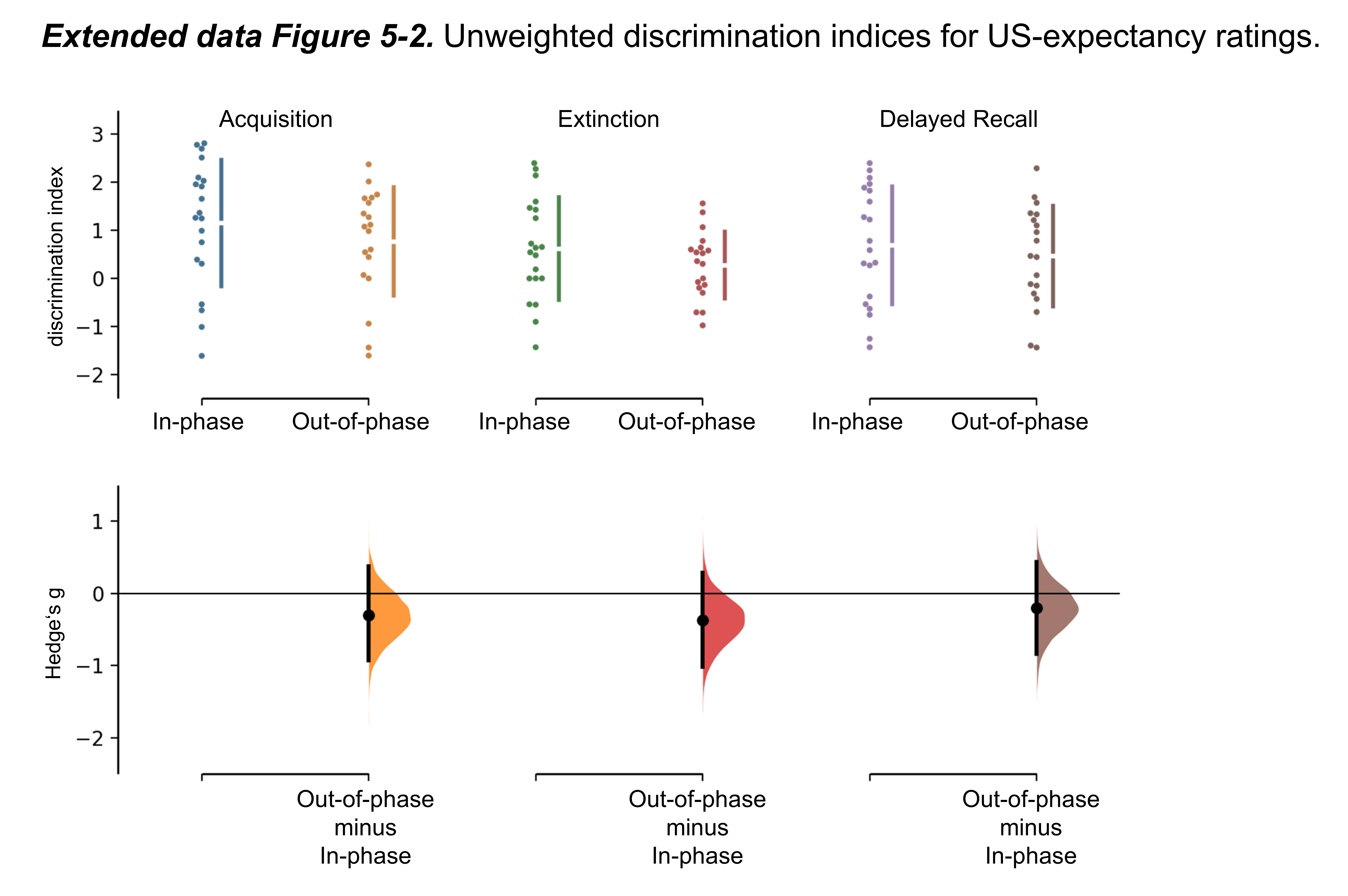

Supplement: Figure 5-2 — Unweighted discrimination indices for US expectancy ratings. US expectancy ratings were z-transformed within each participant using the mean and SD of all US expectancy ratings of a participant. The unweighted discrimination index shown is the difference between ratings of the CS+ and the unweighted average of the four CS– orientations. Data and effect sizes are shown as a Cumming estimation plot (http://www.estimationstats.com). See the legend of Extended Data Figure 5-1 for a detailed description of a Cumming estimation plot. The unpaired Hedge’s g: for acquisition:–0.306 [95% CI, –0.928, 0.375], p = 0.3356; for extinction: –0.372 [95% CI, –1.021, 0.289], p = 0.2346; for delayed recall: –0.198 [95% CI, –0.842, 0.433], p = 0.5166. The 5000 bootstrap samples were taken for CI estimation; the CI is bias corrected and accelerated. The two-sided p values are the likelihoods of observing the effect sizes, if the null hypothesis of zero difference is true. For each permutation p value, 5000 reshuffles of the group labels were performed. Download Figure 5-2, TIF file. [file enu-eN-NWR-0538-20-s04.tif]

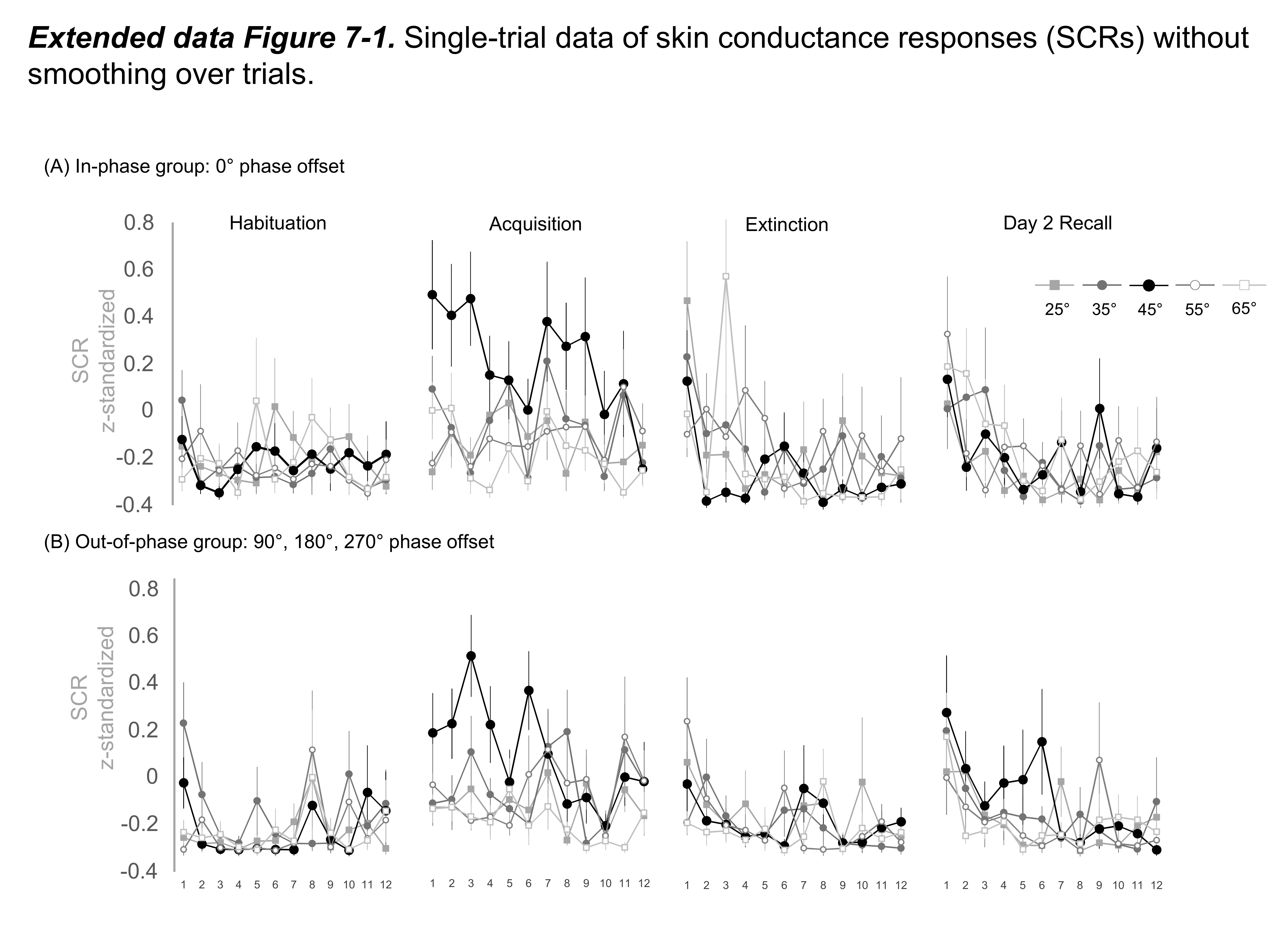

Supplement: Figure 7-1 — Single-trial data of SCRs without smoothing over trials. Same data as in Figure 7, A and B, plotted without the moving average over trials. SCRs are separated by learning phase (habituation, acquisition, extinction on day 1, and delayed recall on day 2) and by the synchronization condition into the in-phase group (i.e., 0° phase offset; A) and the out-of-phase group (i.e., 90°, 180°, and 270° phase offset; B). Error bars show ± 1 SEM. Download Figure 7-1, TIF file. [file enu-eN-NWR-0538-20-s07.tif]

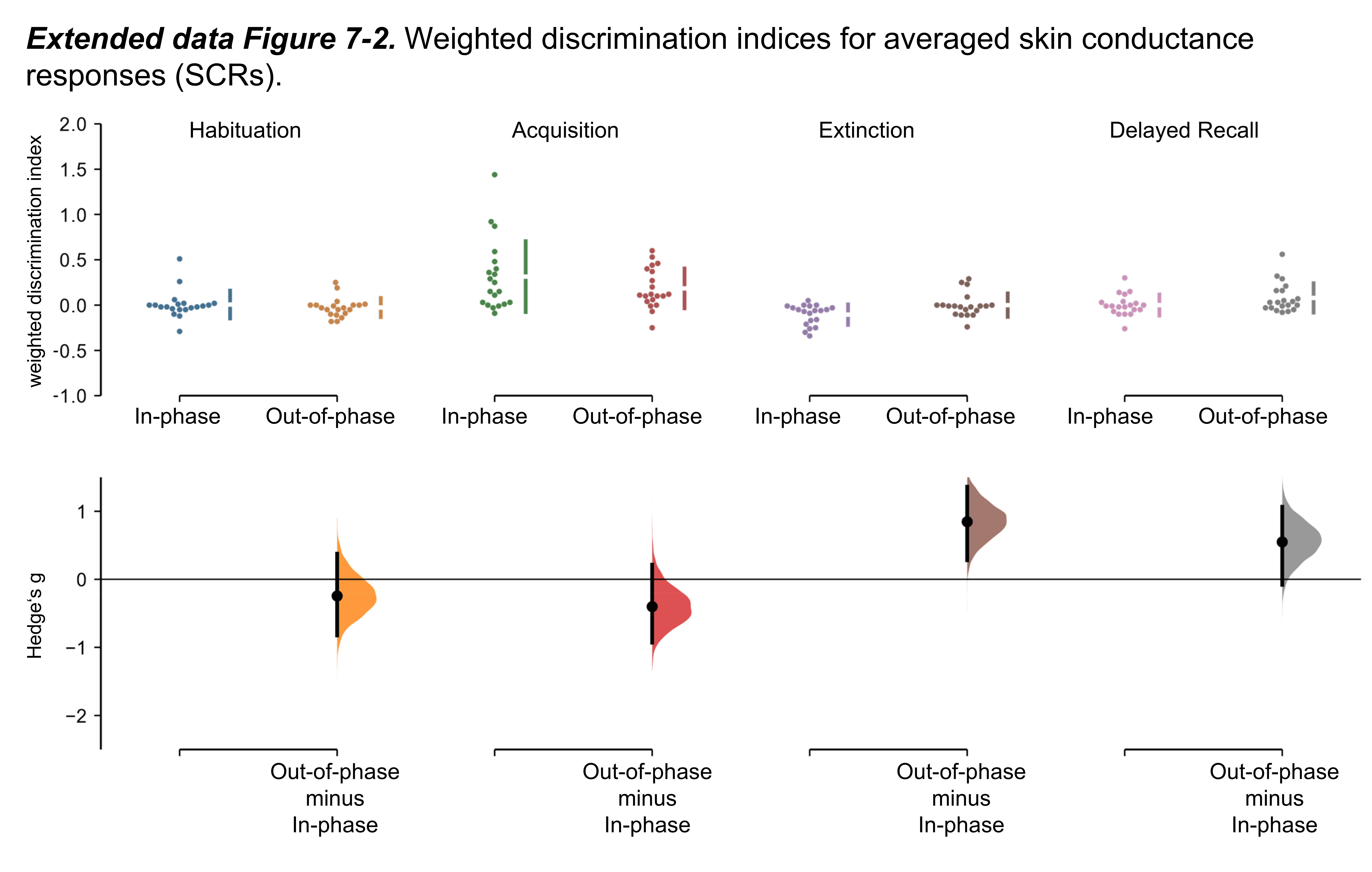

Supplement: Figure 7-2 — Weighted discrimination indices for averaged for averaged SCRs. SCRs were first z-transformed within each participant using the means and SDs over CS and US responses of all learning phases (habituation, acquisition, extinction, delayed recall). With the z-transformed data, we computed a weighted discrimination index per learning phase as the difference between the reinforced 45° (CS+) grating and the weighted average of the four CS– gratings. Weights for the CS– orientations correspond to the angular difference in orientation between the four CS– orientations (25°, 35°, 55°, 65°) and the CS+ orientation (45°): the two more similar CS– orientations (±10° to the CS+) were weighted with 0.33[…], while the more dissimilar orientations (±20° to the CS+) were weighted with 0.166[…]. Data and effect sizes are shown as a Cumming estimation plot (http://www.estimationstats.com). See Extended Data Figure 5-1 legend for a detailed plot description. The unpaired Hedge’s g: for habituation: –0.249 [95% CI, –0.827, 0.371], p = 0.451; for acquisition: –0.405 [95% CI, –0.938, 0.211], p = 0.2044; for extinction: 0.847 [95% CI, 0.277, 1.361], p = 0.0096; for delayed recall: 0.535 [95% CI, –0.091, 1.056], p = 0.0916. Download Figure 7-2, TIF file. [file enu-eN-NWR-0538-20-s08.tif]

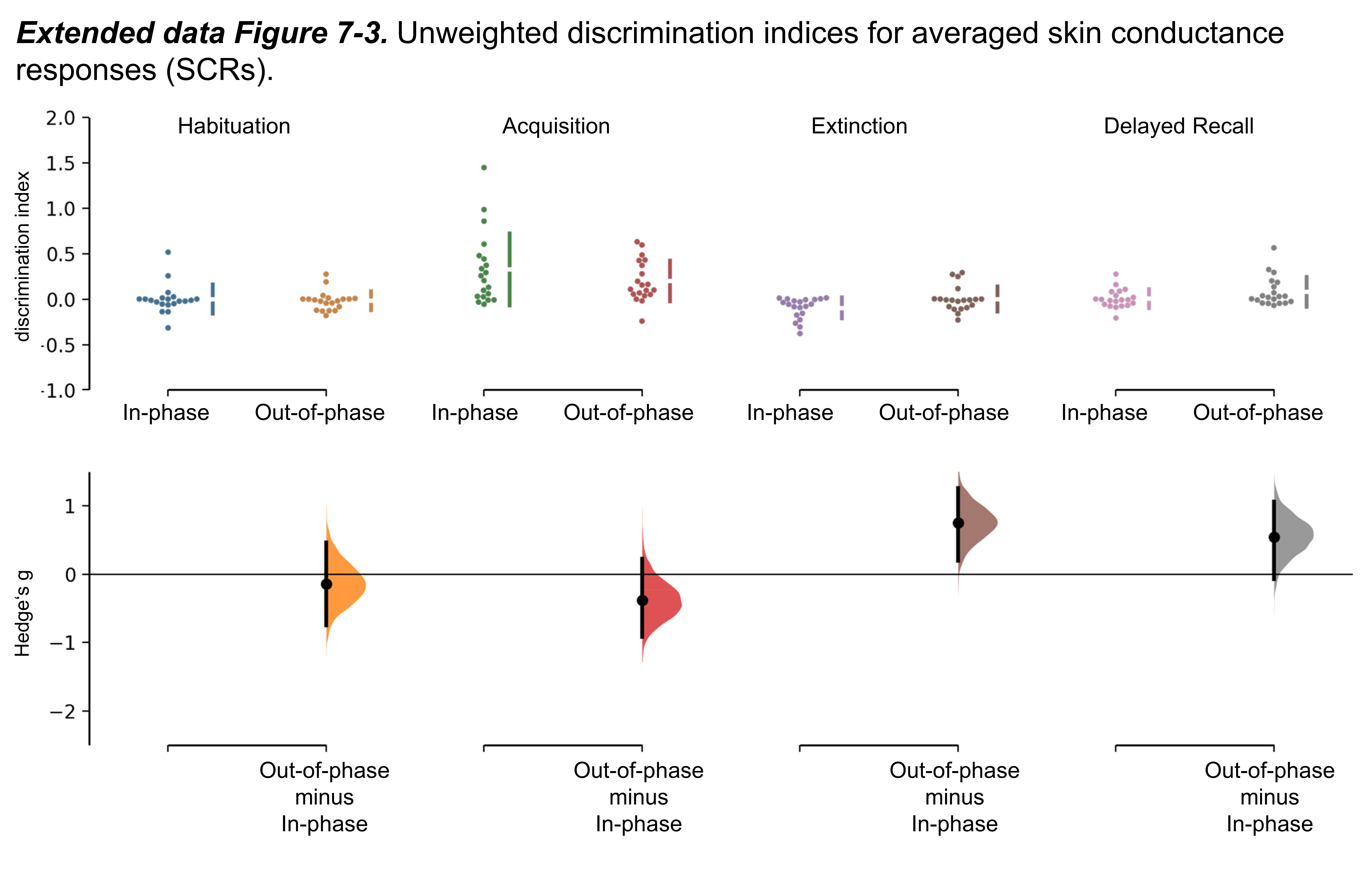

Supplement: Figure 7-3 — Unweighted discrimination indices for averaged SCRs. SCRs were z-transformed within each participant using the means and SD over CS and US responses of all learning phases (habituation, acquisition, extinction, delayed recall). The unweighted discrimination index shown is the difference between SCR to the CS+ and the unweighted average of the four CS– orientations. Data and effect sizes are shown as a Cumming estimation plot (http://www.estimationstats.com). See Extended Data Figure 5-1 legend for a detailed plot description. The unpaired Hedge’s g: for habituation: –0.146 [95% CI, –0.754, 0.461], p = 0.6618; for acquisition: –0.385 [95% CI, –0.920, 0.230], p = 0.2296; for extinction: 0.754 [95% CI, 0.197, 1.259], p = 0.0212; for delayed recall: 0.549 [95% CI, –0.071, 1.059], p = 0.0848. The 5000 bootstrap samples were taken for CI estimation; the CI is bias corrected and accelerated. The two-sided p values are the likelihoods of observing the effect sizes if the null hypothesis of zero difference is true. For each permutation p value, 5000 reshuffles of the group labels were performed. Download Figure 7-3, TIF file. [file enu-eN-NWR-0538-20-s09.tif]

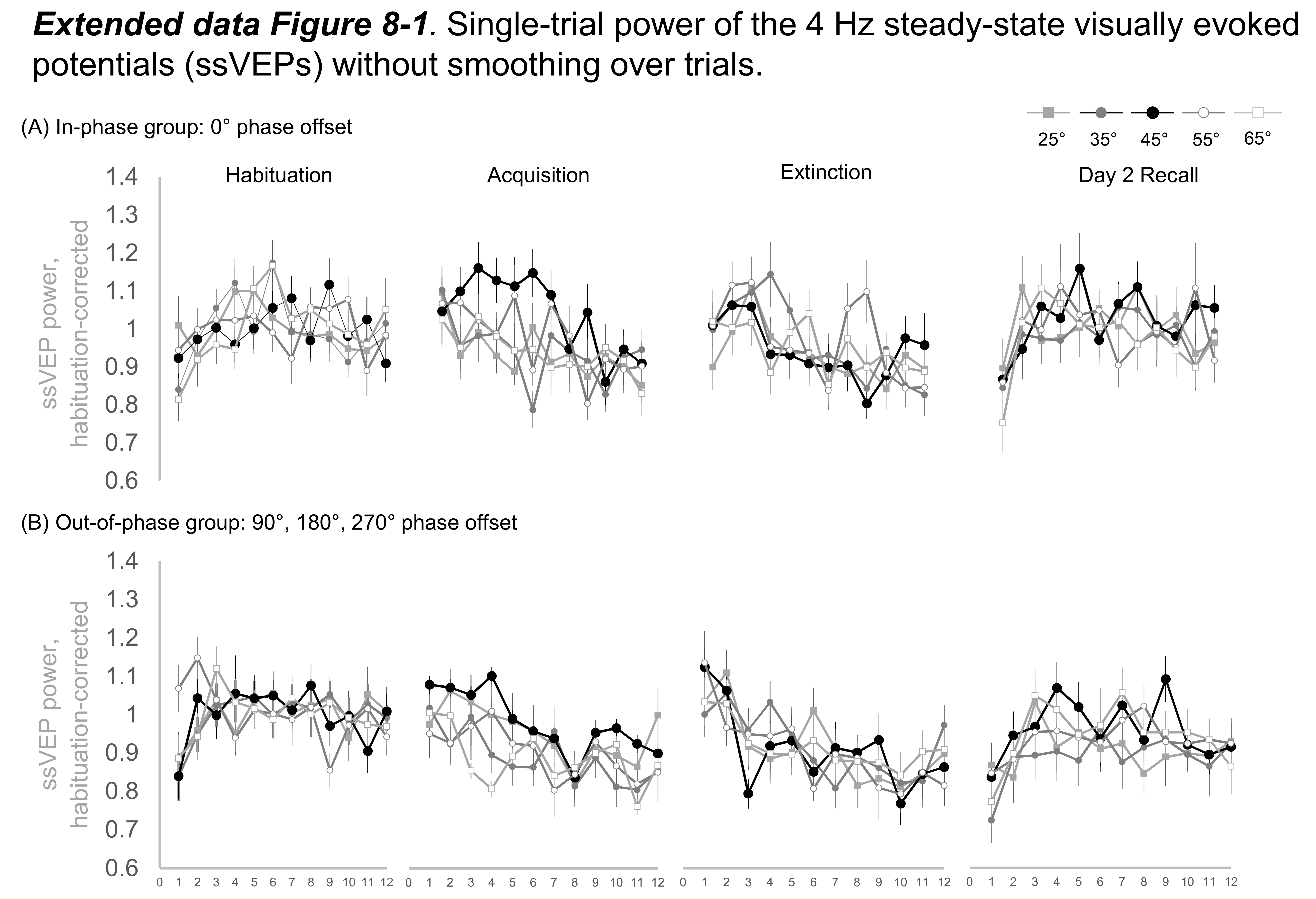

Supplement: Figure 8-1 — Single-trial power of the 4 Hz ssVEPs without smoothing over trials. Same data as in Figure 8, A and B, plotted without the moving average over trials. Single trials are separated by learning phase (habituation, acquisition, extinction on day 1, and delayed recall on day 2) and by the synchronization condition into the in-phase group (i.e., 0° phase offset; A) and the out-of-phase group (i.e., 90°, 180°, 270° phase offset; B). Error bars show ±1 SEM. Download Figure 8-1, TIF file. [file enu-eN-NWR-0538-20-s10.tif]

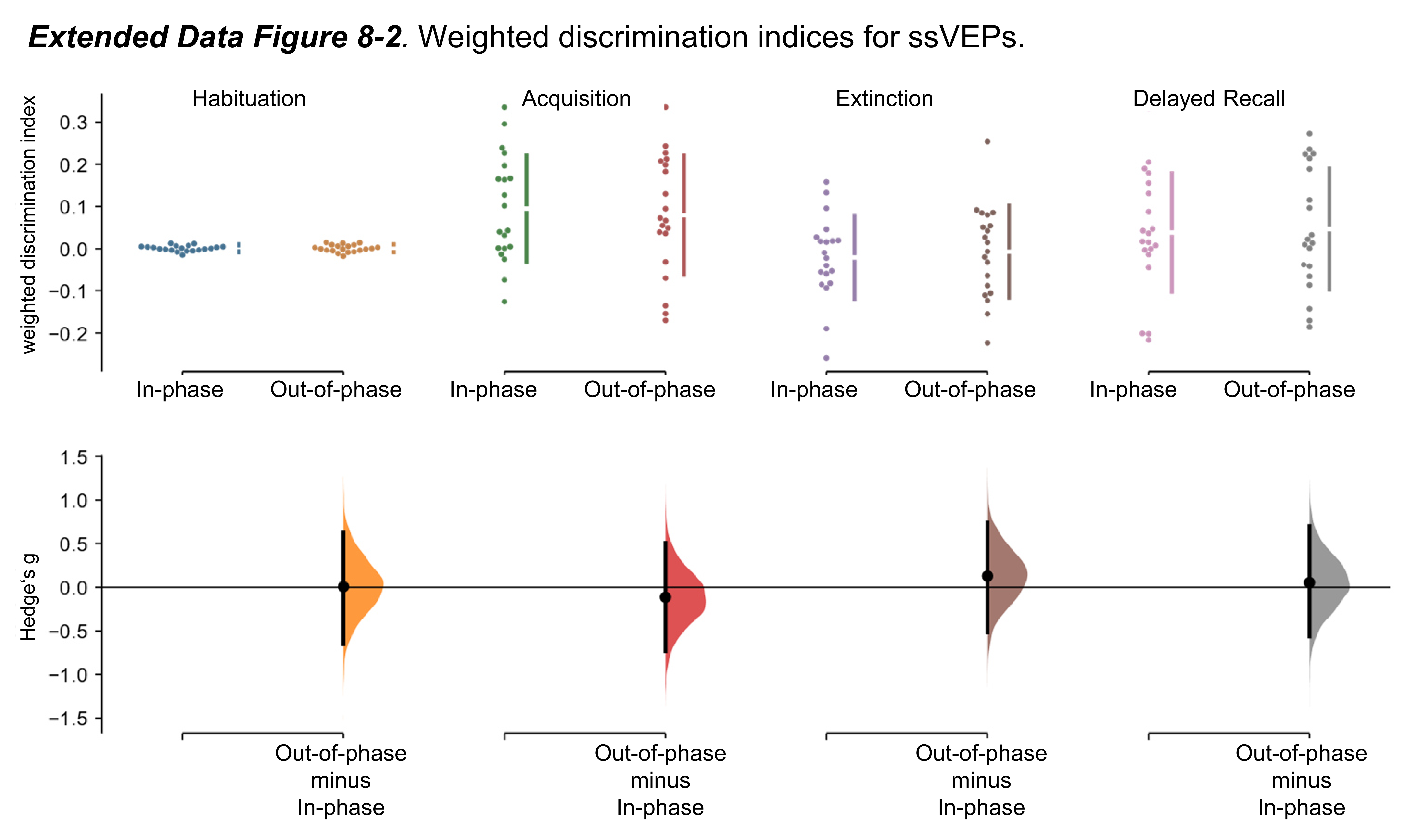

Supplement: Figure 8-2 — Weighted discrimination indices for ssVEPs. Within each learning phase, using the habituation corrected SNR at 4 Hz (Fig. 8C), we computed a weighted discrimination index per learning phase as the difference between the reinforced 45° (CS+) grating and the weighted average of the four CS– gratings. Weights for the CS– correspond to the angular difference in orientation among the four CS– orientations (25°, 35°, 55°, 65°) and the CS+ orientation (45°): the two more similar CS– orientation (±10° to the CS+) were weighted with 0.33[…], while the more dissimilar orientations (±20° to the CS+) were weighted with 0.166[…]. Data and effect sizes are shown as a Cumming estimation plot (http://www.estimationstats.com). See Extended Data Figure 5-1 legend for a detailed plot description. The unpaired Hedge’s g: for habituation: 0.008 [95% CI, –0.652, 0.633], p = 0.979; for acquisition: –0.114 [95% CI, –0.731, 0.511], p = 0.7084; for extinction: 0.130 [95% CI, –0.519, 0.741], p = 0.683; for delayed recall: 0.054 [95% CI, –0.564, 0.702], p = 0.08622. The 5000 bootstrap samples were taken for CI estimation; the CI is bias corrected and accelerated. The two-sided p values are the likelihoods of observing the effect sizes if the null hypothesis of zero difference is true. For each permutation p value, 5000 reshuffles of the group labels were performed. Download Figure 8-2, TIF file. [file enu-eN-NWR-0538-20-s11.tif]

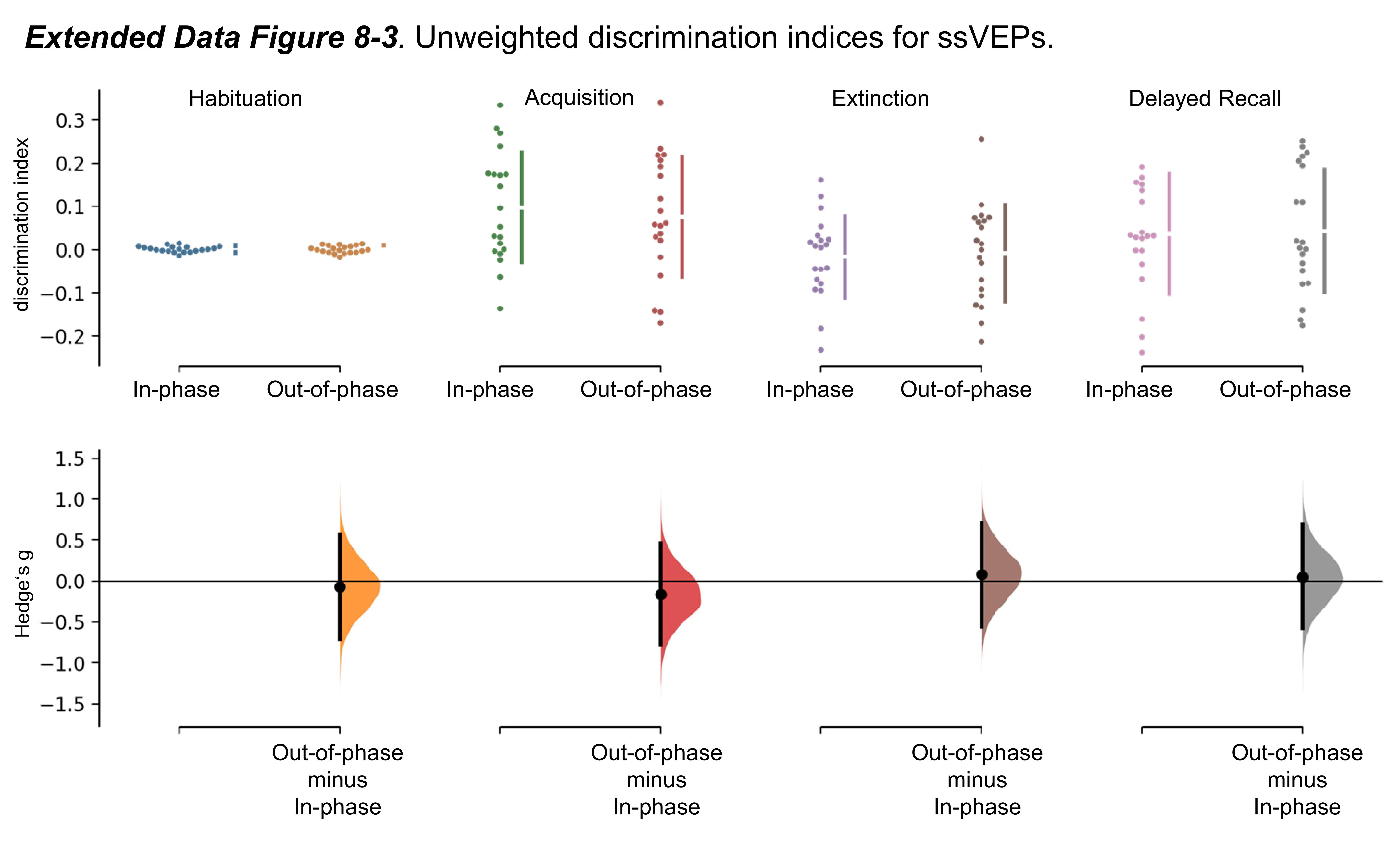

Supplement: Figure 8-3 — Unweighted discrimination indices for ssVEPs. Here, the discrimination index was computed as the difference between the reinforced 45° orientation (CS+) grating and the unweighted average of the four CS– orientations. Data and effect sizes are shown as a Cumming estimation plot (http://www.estimationstats.com). See Extended Data Figure 5-1 legend for a detailed plot description. The unpaired Hedge’s g: for habituation: –0.074 [95% CI, –0.708, 0.569], p = 0.8106; for acquisition: –0.161 [95% CI, –0.774, 0.464], p = 0.6074; for extinction: 0.080 [95% CI, –0.561, 0.706], p = 0.7948; for delayed recall: 0.044 [95% CI, –0.579, 0.687], p = 0.891. The 5000 bootstrap samples were taken for CI estimation; the CI is bias corrected and accelerated. The two-sided p values are the likelihoods of observing the effect sizes, if the null hypothesis of zero difference is true. For each permutation p value, 5000 reshuffles of the group labels were performed. Download Figure 8-3, TIF file. [file enu-eN-NWR-0538-20-s12.tif]
